# Testing the island effect on phenotypic diversification: insights from the *Hemidactylus* geckos of the Socotra Archipelago

**DOI:** 10.1038/srep23729

**Published:** 2016-04-13

**Authors:** Joan Garcia-Porta, Jiří Šmíd, Daniel Sol, Mauro Fasola, Salvador Carranza

**Affiliations:** 1 Institute of Evolutionary Biology (CSIC-Universitat Pompeu Fabra), Passeig Marítim de la Barceloneta, 37-49, 08003, Barcelona, Spain; 2 Department of Zoology, National Museum, Prague, Czech Republic; 3 Center for Ecological Research and Forestry Applications (CREAF), Spanish National Research Council (CSIC), Campus of the Autonomous University of Barcelona, Cerdanyola del Vallès, 08193 Catalonia, Spain; 4 Dipartimento di Scienze della Terra e dell’Ambiente, Università di Pavia, Via Ferrata 1, I-27100 Pavia, Italy

## Abstract

Island colonization is often assumed to trigger extreme levels of phenotypic diversification. Yet, empirical evidence suggests that it does not always so. In this study we test this hypothesis using a completely sampled mainland-island system, the arid clade of *Hemidactylus*, a group of geckos mainly distributed across Africa, Arabia and the Socotra Archipelago. To such purpose, we generated a new molecular phylogeny of the group on which we mapped body size and head proportions. We then explored whether island and continental taxa shared the same morphospace and differed in their disparities and tempos of evolution. Insular species produced the most extreme sizes of the radiation, involving accelerated rates of evolution and higher disparities compared with most (but not all) of the continental groups. In contrast, head proportions exhibited constant evolutionary rates across the radiation and similar disparities in islands compared with the continent. These results, although generally consistent with the notion that islands promote high morphological disparity, reveal at the same time a complex scenario in which different traits may experience different evolutionary patterns in the same mainland-island system and continental groups do not always present low levels of morphological diversification compared to insular groups.

The existence of phenotypically bizarre species in islands – as the Dodo, the Aye-aye or the Galapagos tortoises– has always amazed naturalists and evolutionary biologists. This distinctiveness of so many island species has classically been linked to the impoverished species richness exhibited by islands, consequence of their geographic isolation^1^. This would release new colonizers from competitors and enemies, enabling them the capacity to expand their niches and invade adaptive zones that are normally occupied in the continent^2^. The absence of entire functional groups on islands would even allow some species to evolve toward novel directions and expand the phenotypic space used by their continental relatives^2^. Such an “island effect” would be coupled with high levels of species diversification and accelerated rates of trait evolution, often producing high levels of disparity among island species^3^.

Although the existence of an “island effect” on phenotypic diversification is backed by many empirical examples^2^,^3^, the existence of many instances of island taxa that fail to diversify in insular contexts^4^,^5^,^6^ or that diversify at similar extents to their continental close-relatives^7^,^8^ cautions that this “effect” may not be as general as often assumed. For example, island colonizers may fail to diversify if they do not find sufficient habitat heterogeneity^5^ or if they exhibit structural or functional constrains^9^. Moreover, a number of factors can produce levels of diversification in the continent comparable to those found in islands. Extrinsic factors such as climate change, orogenic processes and episodic mass extinctions can provide novel niches that can potentially trigger high levels of species and phenotypic diversification in continental biotas^10^,^11^. The appearance of key innovations can also facilitate the access to a wider range of novel niches in continental groups, producing patterns of phenotypic diversification similar to those found in islands^12^,^13^.

While more studies are needed to address the generality of the island effect on empirical grounds, a major stumbling block is the need of well-sampled phylogenies in both island and continental domains. The present study uses a completely sampled mainland-island system, the so-called arid clade of *Hemidactylus* geckos^14^ to test whether the patterns of phenotypic diversification in islands actually differ from those occurring in the continent. The arid clade represents a well-studied monophyletic radiation of 48 species distributed across the arid regions of northeast Africa, the Levant, Arabia, the adjoining areas of southwest Asia and, interestingly for our purposes, also in the Socotra Archipelago^14^,^15^,^16^,^17^,^18^ (Fig. 1). This archipelago is located in the northwestern Indian Ocean and originated as a continental fragment that detached from Arabia around 30 Ma^19^. It comprises two main islands, Socotra and Abd al Kuri (with areas of 3,625 km^2^ and 133 km^2^ respectively) and two small islets, Darsa and Samha (5.4 km^2^ and 40 km^2^, respectively), situated 380 km off the southeast coast of Arabia (Yemen), and approximately 100 km east of Somalia off the Horn of Africa (Fig. 1). The *Hemidactylus* geckos of the arid clade are present on all four islands and have reached the archipelago three times independently, two of them producing subsequent intra-island diversification events^16^,^17^. *Hemidactylus* diversity on the small islands of Darsa and Samha is restricted to a single species (*H. homoeolepis*) also present on Socotra^16^,^18^.

The existence of multiple instances of colonization and diversification in the same archipelago provides an opportunity to explore the existence of an “island effect” on phenotypic diversification using independent replicates and in a completely sampled mainland-island system (with a sampling of all phenotypes existing in the continent and in the islands).

To test the “island effect” hypothesis, we began by developing a robust multi-locus phylogeny for the whole arid clade of *Hemidactylus*. We then applied a variety of comparative methods to pose three main questions: 1) Do island and continental species significantly differ in their morphologies, with the most divergent phenotypes occurring on islands? 2) Do insular species assemblages exhibit higher phenotypic disparities compared to continental species assemblages? And if so, 3) Are these higher disparities driven by faster evolutionary rates? Our results yield support for the island effect, but also suggest that the phenomenon is more complicated than often assumed.

## Results

### Phylogeny and ancestral state reconstructions

Sequences of two mitochondrial (*12S* and *cytb*) and four nuclear genes (c-*mos*, *mc1r*, *rag1* and *rag2*) were assembled from previous phylogenetic studies that focused on the arid clade^15^,^16^,^17^,^20^. In addition, we sequenced all six gene fragments listed above for three additional *Hemidactylus* species from the Horn of Africa and Arabia (*H. barbierii*, *H. granosus* and *H. macropholis*) and completed the molecular datasets for 13 species that had *cytb, mc1r, rag2* and *rag1* not previously sequenced.

Our final dataset included the sequences for all 31 species known to occur in Arabia, the Socotra Archipelago, the Levant, and adjoining areas of the Persian region, and 17 species occurring in the Horn of Africa. This dataset is the most complete to date, comprising all known species of the arid clade (a total of 48 species; Supplementary Table S1).

**Figure 2:**
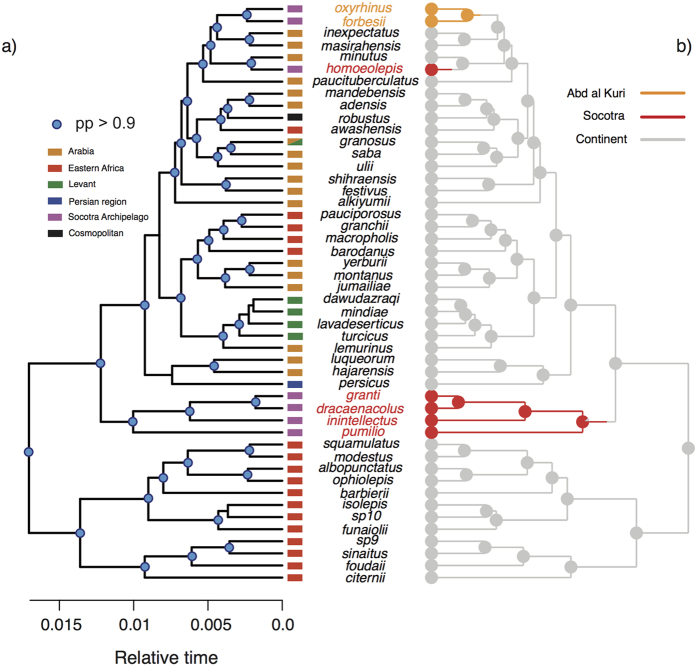
(**a**) Ultrametric tree of the arid clade of *Hemidactylus* geckos derived from the BEAST analysis (summary tree, outgroups not shown). The blue circles indicate nodes with a posterior probability >0.90. The colored rectangles indicate the geographic origin of each species (colors are the same as in Fig. 1). (**b**) Summary tree with the transitions among “continental”, “Abd al Kuri” and “Socotra” (grey, orange and red, respectively) reconstructed according to one possible stochastic character history. The pie charts at the nodes visualize the uncertainty of the ancestral state reconstructions.

The summary tree produced by our phylogenetic analyses recovered 86% of nodes with a posterior probability (pp) higher than 0.90 (high to very high support; Fig. 2a). The phylogenetic relationships depicted by our summary tree (Fig. 2a) were largely consistent with the previous most complete phylogenies of the arid clade of the genus *Hemidactylus*^15^,^16^,^17^.

We assigned lineages to Socotra Island, Abd al Kuri Island or mainland by means of 1,000 stochastic ancestral reconstructions computed on the summary tree and one reconstruction computed on each of the 1,500 trees sampled from the posterior distribution (see material and methods).

The stochastic state reconstructions over the summary tree revealed negligible rates of transition from islands to the continent (q < 10^−5^), and high rates of transitions from the continent to Socotra and Abd al Kuri (q = 9.00 and 4.26, respectively). Similar values were computed in those reconstructions involving each of the 1,500 trees (results not shown). In both summary tree and in the set of 1,500 trees, most of the stochastic reconstructions placed all nodes splitting island lineages within island categories, suggesting intra-island diversification (Fig. 2b). This is congruent with previous dating estimates, which dated all intra-island nodes well after the detachment between Arabia and the Socotra Archipelago^16^.

### Differences in morphology and disparity between island and continental groups

We used body size and head proportions to morphologically characterize the species in the arid clade. Body size subsumes much of the animal’s biology^21^,^22^ and has commonly been found to diverge after island colonization^23^,^24^,^25^. Head shape may also diverge after island colonization^26^ given its role in multiple and highly relevant ecological activities in lizards, including feeding, refuge use, mating and aggressive interactions^27^,^28^,^29^.

**Figure 3:**
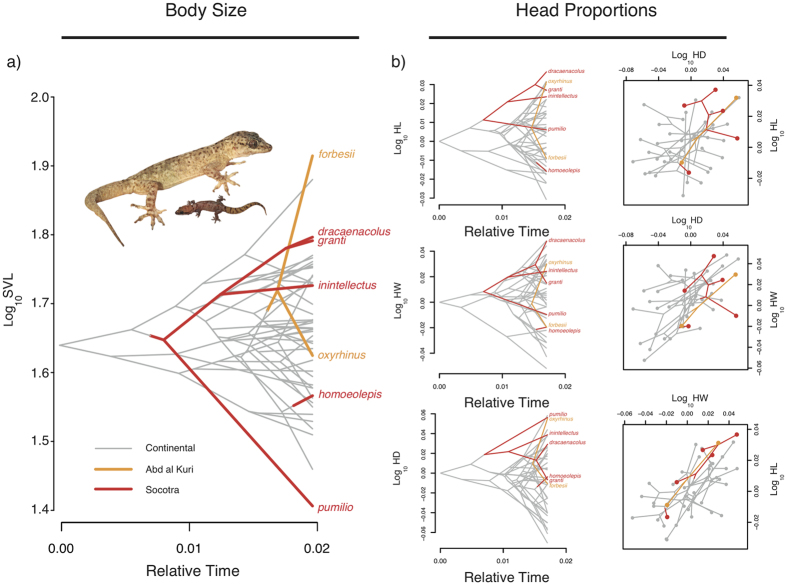
(**a**) Phenogram showing the body size variation across the 48 taxa that form the arid clade of *Hemidactylus* geckos. The vertical position of nodes and tips represents the known or estimated body size (log_10_-transformed SVL, snout-vent length), while the horizontal position reflects relative time. The lineages are highlighted in grey (for the continent), orange (for Abd al Kuri) and red (for Socotra). The pictures show the biggest and the smallest species (*Hemidactylus forbesii* from Abd al Kuri and *H. pumilio* from Socotra) at the same scale. (**b**) Visualization of the variation in head proportions for the 42 species for which head dimensions were obtained. The left panel represents the phenograms of head dimensions. The right panel visualizes the insular and the continental phylomorphospace (HD: head depth; HW: head width; HL: head length). In all panels geckos are highlighted in grey (for continental species), orange (Abd al Kuri species), and red (Socotra species). Pictures by Roberto Sindaco.

Island species attained the most extreme sizes of the entire arid clade, *H. forbesii* (from Abd al Kuri) being the largest and *H. pumilio* (from Socotra) being the smallest (Fig. 3a). These two size extremes were the consequence of substantial amounts of size change associated with two of the intra-island speciation events, the single split within Abd al Kuri separating *H. forbesii* and *H. oxyrhinus* and the most basal split within Socotra separating *H. pumilio* from the remaining Socotran species. Aside from these two size extremes, the sizes of the rest of island species were within the range observed in the continent (Fig. 3a).

The variation in head proportions was roughly distributed along a “short-narrow-low” to “long-wide-high” continuum of head proportions, and most island species presented head proportions within the range observed in the continent (Fig. 3b).

**Figure 4:**
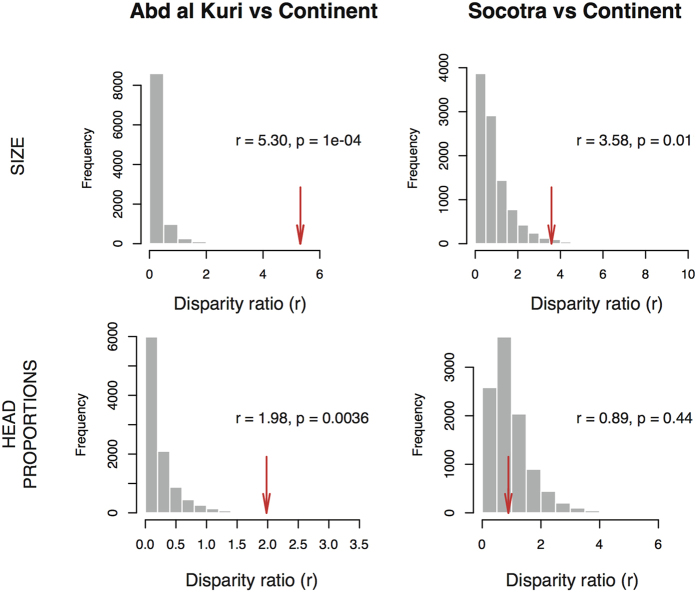
Ratios of island versus continent in body size and in head proportions disparitiy (r). The empirical values (arrows) are given with the *p*-values calculated by means of 10,000 Brownian motion simulations computed on the summary tree. The distributions of simulated values are represented by grey bars.

In agreement with the observed morphological overlap, the results of the phylogenetic ANOVA and MANOVA (comparing insular and continental morphologies) showed no significant differences in size or head proportions between island and mainland clades, regardless of whether islands were grouped together or not, and whether the analyses were performed on the summary tree or on each of the 1,500 trees (*p*-values > 0.05 in all cases, Supplementary Table S3). Nevertheless, body size disparities for Socotra and Abd al Kuri were respectively 3.58 to 5.30 times the mean disparity of the continent, and were significantly higher than those expected by the null distribution produced on the summary tree (with *p*-values computed at 0.01 and 10^−4^ for Socotra and Abd al Kuri, respectively) (Fig. 4) and on the set of 1,500 trees (with *p*-values ranging from 0.004 to 0.007 and from 0.0014 to 10^−4^ for Socotra and Abd al Kuri, respectively). Moreover, the resampling of 10,000 random continental sets of two and five species simulating the communities of Abd al Kuri and Socotra, respectively, did not produce the ranges of body size variation observed in the islands (*p*-values < 0.05; Supplementary Fig. S1).

Regarding head proportions, only Abd al Kuri produced disparities significantly higher than the random expectation (*p*-value on the summary tree computed at 0.0036 and *p*-value on the 1,500 trees ranging from 0.01 to 0.001). In this case, disparities were approximately twice the observed disparity in the continent when the summary tree was used (Fig. 4) and from 1.1 to 2.8 times the continental disparity when the analysis was conducted on the 1,500 trees. However such disparity was not observed when the ranges of all head variables in the islands were compared to the ranges of 10,000 sets of two and five species resampled from the pool of continental species. In this case, head variables failed to show wider ranges of variation compared to continental communities (all *p*-values > 0.05; Supplementary Fig. S1).

### Testing for differences in tempos of phenotypic diversification

To study whether insularity was associated with accelerated rates of morphological evolution, for body size and for each of the three dimensions of head proportions, we compared the fitting of three alternative models of evolution: BMS, a Brownian motion model that assumed that the lineages in Socotra, Abd al Kuri and mainland evolved according to different Brownian rate parameters (σ^2^), BM1, a Brownian model that assumed a single σ^2^ across all the lineages in the phylogeny and OU1, an Ornstein-Uhlenbeck model that assumed, in addition to a single σ^2^, the existence of a global phenotypic optimum (θ) and a rate of adaptation towards it (α)^30^,^31^,^32^.

For head proportions, the three models (BMS, BM1 and OU1) were equally supported (with differences between mean AICc values always <5 units between each other) except for HD, in which OU1 was the most supported model (with a difference of 9.8 units between the mean AICc of OU1 and the mean AICc of the second most supported model, Supplementary Fig. S2). Overall this yields no indication that the rate of head shape evolution varied between islands and mainland.

**Figure 5:**
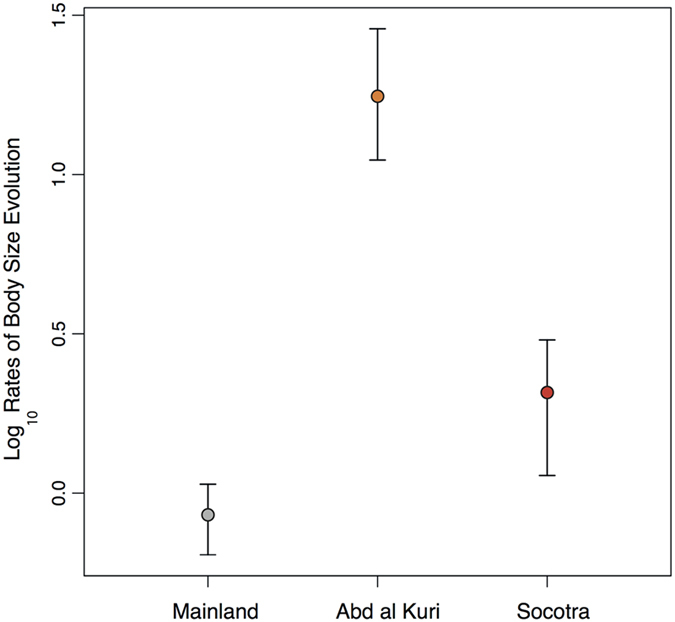
Plot of the relative rates (at log_10_-scale) of body size evolution and its associated 95% confidence intervals for each geographic area, estimated according to BMS model. This model allows lineages in Socotra, Abd al Kuri and in the continent to evolve at different rates. The rates shown are based on the set of 1,500 trees (results for the summary tree were very similar).

For body size, however, the model assuming a heterogeneous rate among Abd al Kuri, Socotra and continent (BMS) was always the most supported one (with a difference of 11 units between the mean AICc of BMS and the mean AICc of the second most supported model, OU1, Supplementary Fig. S2). The visualization of the rate estimates and their confidence intervals was consistent with a pattern of extreme rate heterogeneity. The clade of Abd al Kuri presented mean rates of body size evolution more than 20 times higher than the mean rate observed in the continent, and more than eight times the rates computed for Socotra. The rates in Socotra, although high, were only around two times the mean rates of the continent (Fig. 5). Parametric bootstrapping also supported the BMS model for body size evolution, with *p*-values ranging from 0 to 0.019 for the summary tree and from 0 to 0.04 in the posterior distribution of trees. Our estimates of statistic power ranged from 92 to 99% for the summary tree and from 84 to 99% for the posterior distribution of trees (Fig. S3).

**Figure 6:**
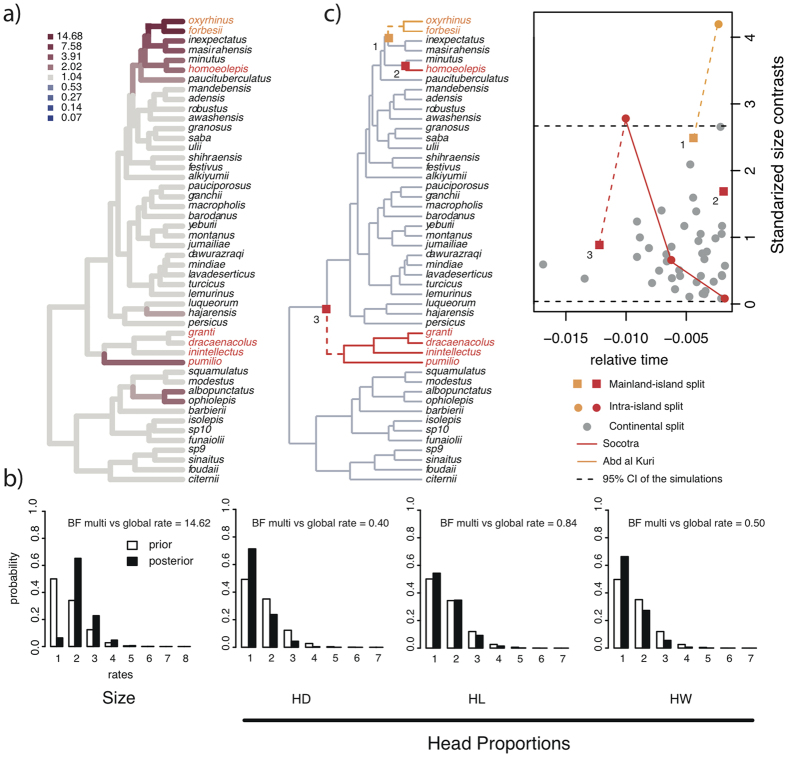
(**a**) Summary tree with branches colored to reflect the magnitude of shifts in the rates of body size evolution as computed by the “auteur” model. Background rates (those not deviating from the median rate across the tree) are colored light gray; those branches with rates higher than background rates are colored in red and their intensity varies in proportion to their rate value. (**b**) Distributions of the prior and posterior values of rate heterogeneity with the estimates of Bayes factors comparing a multi- vs single-rate pattern of phenotypic evolution (HD: head depth, HW: head width; HL: head length). (**c**) Distribution of the standarized body size contrasts across time. The colors and numbers in the plot match with those in the tree on the left. The dashed lines indicate the 95% CI of 10,000 simulations generated assuming a single rate parameter across the tree.

We also estimated rates of phenotypic evolution along branches of the phylogeny without *a priori* specifying which lineages of the tree were assigned to an island or a continental domain. Our results with this approach (using “auteur”, see material and methods) confirmed the superiority of the multi-rate scenario for body size relative to the single-rate scenario (BF = 14.62) (Fig. 6b). This was further confirmed by the set of 1,500 trees, which produced similarly high BF values for most of the trees (mean BF comparing multiple *vs* single rates = 30.24). In the summary tree and most of the 1,500 trees, the comparison between the prior and posterior rate distributions revealed a two-rates situation as the pattern of rate heterogeneity with the highest posterior probability (Fig. 6b). The clade formed by *H. oxyrhinus* and *H. forbesii* was in 99% of cases associated with the rate shift presenting the highest posterior probability, always in the direction of increasing rates of body size evolution. When the posterior rates were visualized on the branches of the summary tree, the lineages of Abd al Kuri presented the highest computed rates but, remarkably, these appeared to be nested within a continental clade with high rates of size evolution (which also included the Socotran *H. homoeolepis*). Aside from *H. homoeolepis*, the only Socotran lineage with accelerated rates was *H. pumilio*, with the rest of the Socotran species presenting rates in the range of most continental species (Fig. 6a).

Finally, to investigate how the heterogeneity in the rates of body size evolution was structured along with time, we computed the absolute values of the standardized independent contrasts (another proxy for Brownian rates) and visualized them through time. This approach showed how the intra-island split in Abd al Kuri and the first intra-island split of Socotra were the first and the second most extreme contrast values respectively (Fig. 6c), a pattern consistent with all previous analyses. These contrast values were significantly higher than the null distribution of 10,000 simulations based on a single rate parameter across the tree (*p*-values < 0.05), highlighting their extreme magnitudes. The splits that followed the onset of diversification in Socotra presented decreasing contrast values, a pattern consistent with slowing rates of body size evolution as the intra-island diversification proceeds (Fig. 6c). Although most of the continental lineages presented very low rates of body size evolution, we detected an upsurge in the rates of body size evolution toward recent times, in some cases attaining values similar to those found in the earliest speciation event in Socotra (Fig. 6a–c).

## Discussion

Our results provide strong evidence supporting an acceleration in the rates of body size evolution that produced great disparities in Abd al Kuri and, to a lesser extent, also in Socotra. However, our analyses also detected high rates of body size evolution in certain continental clades and failed to detect any difference in the rates of evolution of head proportions between islands and the continent. Thus, although our results generally support the existence of an “island-effect” on morphological diversification, at the same time they reveal a complex scenario in which neither all traits respond in the same way to island colonization nor all continental clades present low levels of phenotypic diversification compared to island clades.

Body size is the measured trait that experienced the greatest disparity in the Socotra Archipelago. This lines up with previous studies that highlight body size as one of first traits to diverge after island colonization^2^,^33^,^34^. These examples of extreme size diversification in islands are consistent with the notion that after island colonization groups are able to expand their trait variation in response to novel ecological opportunities^2^,^3^. In the arid clade, this phenotypic expansion was beyond the limits of size variation existing in the continent, with island clades producing the largest and the smallest sizes of the radiation.

The existence of size extremes on islands has been detected in many instances^13^,^25^,^35^ and it is usually explained as a consequence of the absence of certain competitors or predators which otherwise would have limited such extreme sizes^36^,^37^. For instance, in many insular systems lacking mammal predators reptiles often produce gigantic species^38^, including the biggest living gecko in the world (*Rhacodactylus leachianus*)^39^. The absence of predators (typically predatory mammals or snakes) has been hypothesized to promote gigantism by allowing an increase of foraging time and enabling lizards to take the role of top predators^36^,^38^. Similarly, in the case of the Abd al Kuri, the complete absence of native terrestrial mammals and snakes could also have contributed to produce the biggest size in the arid clade. However, in the Socotra Archipelago the size shift not only goes towards gigantism (in Abd al Kuri) but also to dwarfism (in Socotra).

The fact that size shifts occur in opposite directions on both islands can be explained by intrinsic or extrinsic mechanisms. Intrinsically, different clades may be differently predisposed to dwarfism or gigantism^40^. Yet this does not seem the case, at least for the island of Socotra, where *H. pumilio* is the sole representative of small sizes in an otherwise big sized clade (Fig. 3). Alternatively, the ecological context may determine the tendency to evolve towards bigger or smaller sizes^41^. For instance, although native mammals are absent from both Socotra and Abd al Kuri, the presence of snakes in Socotra could have limited the evolution of big sizes on this island but not on Abd al Kuri where snakes are not present^18^.

Aside from the effect of the lack of predators, the absence of certain lizard groups in the archipelago could also have contributed to produce the observed size extremes. Other nocturnal geckos, like the genera *Ptyodactylus* of very big size, and *Tropiocolotes* of very small size, known to occupy similar niches to *Hemidactylus* and probably competing in mainland Arabia^42^, are not present in the Socotran Archipelago. This absence of competitors could have allowed *Hemidactylus* the possibility to attain sizes in the archipelago not reachable in the continent due to niche pre-emption.

The great size disparity observed in the Socotra Archipelago was also associated with accelerated rates of body size evolution, although these rates differed in magnitude depending on the island. Abd al Kuri presented the highest rates computed in the arid clade, while in Socotra the rates were substantially lower (Fig. 5). The observed differences in rate between islands, with species in Abd al Kuri evolving eight times faster than in Socotra, could be explained by the differences in island area. Indeed, models taking into account optimal body mass and allometric scaling laws, predict that the rate and the magnitude of body-mass change is inversely proportional to island area^43^. Such relation has been empirically probed in insular mammals^44^ and could possibly apply to the case of the Socotra Archipelago (Abd al Kuri being 27.25 times smaller than Socotra). However, this rate difference could also reflect the different ages of the onset of diversification in the two islands. The diversification in Abd al Kuri is very young (from 1 to 5 Ma^16^) and is likely under an “early burst” dynamics, when rates of phenotypic evolution tend to be very accelerated^3^,^45^. This contrasts with the much older radiation in Socotra (from 8.8 to 21.5 Ma^16^) that has experienced a progressive decrease in diversification rate, therefore lowering the mean rates in the Socotran clade (Fig. 5).

As for the mechanisms that produced the observed size divergences, these may vary from island to island. The small size of Abd al Kuri along with its low physiographic complexity makes conceivable phenotypic differentiation and speciation in sympatry. In a plausible scenario, the extreme body size divergence exhibited by the two sister species (Supplementary Fig. S4) could be the consequence of disruptive selection driven by intraspecific competition. Indeed, a common consequence of the release from interspecific competitors and predators is an increase of intraspecific competition in island populations^37^,^46^. In such situations, disruptive selection may act if the intermediate and more common phenotypes, e.g. those of intermediate size, face a stronger competition than those at the tails of the size distribution. This results in lower fitness of the intermediate phenotypes leading to an expansion towards new and less exploited resources, driving a great phenotypic divergence and ultimately leading to ecological speciation^47^. Such an explanation is appealing, as it would account for both the *in situ* speciation event, and for the large degree of size disparity occurring in the island. While speciation likely occurred sympatrically in the smaller island of Abd al Kuri, for the larger and topographically complex Socotra Island, allopatric speciation cannot be excluded, coupled with size divergence after secondary contact.

In both scenarios, resource partitioning would be the driver of body size divergence, as it has been proposed in many other insular taxa with extreme size disparities^48^,^49^. In fact, the distribution of the species in the Socotra Archipelago provides additional evidence supporting a scenario of size-mediated ecological divergence. All species substantially differing in size are found in sympatry, while the species of similar sizes (e. g. the sister species *H. dracaenacolus* and *H. granti*) always occur in strict allopatry^18^. The non-coexistence between species overlapping in size can be interpreted as a signature of size-based resource partitioning, as it has been shown in prior studies^34^. Another pattern consistent with a scenario of size-mediated resource partitioning is that, following the innermost split in Socotra, the clade experienced a rapid slowdown in the rates of body size evolution in subsequent diversification events (Fig. 6c). This is a pattern widely detected in many examples of adaptive diversification^3^ and it often reflects an increasing saturation of the niche space (decline of ecological opportunity) as a clade acquires more species during the course of its radiation^45^. Additional empirical evidence, e. g. data on the diets of the different species, would be required to confirm this scenario of size-based resource partitioning.

Regarding continental geckos, most of them converged into similar intermediate sizes with low disparities, a situation expected if high levels of competition and predation constrain the morphospace in the continent. Aside from this, the much larger continental area may potentially accommodate multiple allopatric species with similar morphology and ecology, further contributing to the low levels of disparity observed in continental communities^35^. Yet, according to our results on the temporal dynamics of body size diversification, continental clades experienced an upsurge in the rates of disparification in recent times (Fig. 6). This is associated with some lineages that have recently radiated in southeastern Arabia, which exhibited accelerated rates of body size evolution compared to the rest of the continental lineages and attained rates comparable in magnitude to those computed for the onset of the diversification in Socotra (Fig. 6c).

Therefore, the dynamics of phenotypic diversification in mainland-island systems seems more complex than often assumed. On one hand, the fact that the Abd al Kuri clade (the one that experienced the greatest levels of body size diversification) is also nested in a group of continental species with high rates of size diversification, suggests that pre-adaptations or dynamics of high diversification acquired in continental settings may produce synergies with the ecological opportunity offered by islands and may also contribute to the patterns of phenotypic disparity observed in insular environments. On the other hand, the mere existence of continental clades exhibiting accelerated rates of body size diversification invites to re-consider an implicit assumption of the “island-effect”, i.e. that mainland communities are often ecologically saturated and always tend to present low levels of diversification compared to islands^2^.

Finally, regarding head proportions, our results failed to detect any significant difference between mainland and island species in terms of morphology or in the rates of evolution. Disparities of head proportions were significantly greater than the continent only in Abd al Kuri. These results contrast with previous studies in lizards that found significant differences in head shape between mainland and island populations^26^. One possible explanation is that in the arid clade, the difference in head proportions might not be as important for resource partitioning as the difference in body size, so that coexisting species might overlap in head proportions as long as they diverge in size.

Another possibility is that ecological or functional constrains could potentially limit high levels of head shape disparity^50^. In this regard, the existence of a global optimum for head depth could be consistent with such scenario and agrees well with the need of dorsoventrally compressed bodies and heads of many species of lizards (and many geckos) that use crevices as retreats from predators^15^,^51^. Additional data on head morphology combined with diet composition and ecology would shed light on this matter.

In conclusion, our analyses yield some evidence for the theoretical expectations of enhanced phenotypic disparity and of highly accelerated rates of diversification in islands compared to continents. However, our results also draw a much more complex picture in which not all islands attain similar amounts or rates of disparification, and in which the island effect is not equally detected in all phenotypic traits. Moreover, some relatively young continental clades also seem to exhibit rates of phenotypic diversification similar to those observed on islands. Such a complex picture is consistent with the view that the “island effect” does not reflect any fundamental difference between islands and continents but rather a difference in the likelihood of the ecological space being unsaturated^2^, and of some functional groups being absent. These conditions influence the ecological opportunities and the consequent evolutionary diversification of the new colonizers. As geographic isolation increases the likelihood that islands are unsaturated^2^ and functionally disharmonic, some differences in disparity and evolutionary rates are expected even when the processes acting on the islands and in continents are not qualitatively different. These differences should be particularly notorious in organisms with limited dispersal abilities, like our geckos, in which evolution *in situ* plays a major role in niche partitioning.

## Methods

### Ethic Statement

Most part of the investigated material comes from museum voucher specimens (BMNH London, CAS San Francisco, IBE Barcelona, NMP Prague; see Supplementary Table S2). Vouchers and tissue samples were kindly accessed as loans by the appropriate curators with their permission to use the samples for DNA analyses (B. Clarke and E. N. Arnold – BMNH; J. Vindum – CAS; S. Carranza – IBE; J. Moravec – NMP). The remaining samples were obtained in the field with appropriate collecting permits (Oman: issued by Ali Alkiyumii, Ministry of Environment and Climate Affairs of the Sultanate of Oman: refs 08/2005, 16/2008, 38/2010, 12/2011; Yemen: issued by Omer Baeshen, Environment Protection Agency, Sana′a, Republic of Yemen: Ref 10/2007; Kenya: issued by National Council for Science and Technology (NCST), Nairobi, Kenya). No endangered or protected species were collected and no samples from protected or private areas were used for this study. Research was carried out in accordance with the guidelines of Central Commission for Animal Welfare of the Czech Republic, which also approved the methodology used to capture and handle all animals used in this study (accreditation no. 1090/2012–MZE–17214). All efforts were made to minimize animal suffering.

### Phylogenetic analysis and ancestral reconstructions

Primers and conditions used for the amplification and sequencing of the different fragments followed methods described in ref. 52. DNA sequences were aligned using MAFFT v.6 53 with the options “maxiterate 1000” and “localpair”. Poorly aligned positions in the *12S* marker were eliminated by means of G-blocks^54^, using low stringency options. The final alignment consisted of 4,016 bp as follows: 379 bp of *12S*; 1,137 bp of *cytb*; 402 bp of c-*mos;* 666 bp of *mc1r*; 1,024 bp of *rag1;* and 408 bp of *rag2*. Best fitting nucleotide substitution models were selected for each partition under the Akaike information criterion (AIC)^55^, using jModeltest v.0.1.1^56^. The best models were GTR + I + G for *12s*, *cytb* and *mc1r*, and TrN + G for c-*mos*, *rag1* and *rag2*. Alignment gaps were treated as missing data and the nuclear gene sequences were not phased.

After the alignment of our molecular dataset, phylogenetic analyses were performed using package BEAST v1.6.2 57 (see Supplementary Material for more information on the BEAST analysis). A summary tree was calculated as the maximum clade credibility tree with median node heights and, to incorporate phylogenetic uncertainty into our comparative analyses, we resampled the posterior distribution of trees resulting from our BEAST analysis to obtain a sample of 1,500 trees that varied in topology and branch lengths.

Ancestral state reconstructions were conducted using the function “make.simmap” from the R package “phytools^58^”. This function essentially fits a continuous-time reversible Markov model (in our case allowing different transition rates between all states) and simulates plausible stochastic character histories along the tree using the most likely model in combination with the states assigned to the tips of the tree.

### Phenotypic data collection

Body size was measured as the snout-vent length (SVL) with a calliper to the nearest 0.1 mm. We measured SVL for 692 specimens belonging to 46 species. For two additional species, *Hemidactylus modestus* and *H. funaiolii*, SVL could be obtained from the original species descriptions (Supplementary Table S2). Head shape was measured as its length (from the tip of the snout to the retroarticular process of the jaw), width (widest part of the head) and depth (maximum depth of the head). Measurements of adult specimens only were taken with a calliper to the nearest 0.1 mm for 684 individuals belonging to 42 species (91.3% taxon sampling; see Supplementary Table S2 for details on the specimens measures as well as the museums and collections visited to assemble the dataset). Given that preliminary analyses showed no significant differences between males and females in any of the measurements taken (see also^18^), both sexes were pooled together.

To remove the effect of body size from the head variables, we computed the residuals of a regression of each variable against SVL (both log_10_ transformed). This was done using the species means and correcting by the expected phylogenetic covariances among species^59^. As a way to accommodate phylogenetic uncertainties in the computation of residuals, these were recomputed for each different topology tree in the set of 1,500 randomly sampled trees. We therefore produced 1,500 sets of residuals of head dimensions that were subsequently fed in all subsequent analyses.

### Exploration of morphological variation in island and mainland geckos

We used the function “phenogram” in the R package “phytools^58^” to visualize the variation in size and head proportions across the summary tree. This function essentially projects the phylogeny into a space defined by the phenotype (on the y axis, including the values at the tips and the values reconstructed at the nodes) and time (on the x axis). Given the multivariate nature of the head proportions, we also visualized its variation by means of the “phylomorphospace”. In this case the tree is projected into a bivariate space represented by the species values and the reconstructed states at the nodes for each combination of two head variables^60^. Both representations allow the identification of the major trends of phenotypic change across the tree, as well as the different magnitudes of variation (proportional to the vertical component of each branch in the “phenogram”, and proportional to the branch lengths in the “phylomorphospace^60^”).

To formally assess whether mainland and island species differed in morphology, we performed a phylogenetic ANOVA and MANOVA on size and head proportions respectively. Significance of the empirical *F* statistic (for ANOVA) and the *Wilk’s lambda* (for MANOVA) was assessed by means of null distributions based on 10,000 Brownian motion simulations. For body size, these simulations were based on a maximum likelihood (ML) estimate of the empirical rate parameter. For head proportions, simulations were based on the ML estimate of the evolutionary variance-covariance (vcv) matrix. Both analyses were performed in the R package “geiger^61^” and were conducted on the summary tree and on the set of 1,500 trees.

### Testing for differences in phenotypic disparities between islands and mainland

We assessed the differences of disparity between mainland and island settings in two different ways. We first compared the range of body size and variation in head proportions (maximum - minimum value) existing in the two islands with 10,000 randomly assembled “pseudo-communities” of two and of five continental species, thus simulating the two species existing in Abd al Kuri and the five in Socotra. In this way we assessed whether any combination of species existing in the continent produced the range of phenotypic variation existing in the islands.

Next, we compared island versus continental disparity incorporating the phylogeny in the analysis. To do this, we first defined disparity as the average squared Euclidean distance computed between the sizes and head proportions of all pairs of species coexisting in a given area^35^. In this way we calculated the disparity in body size and head proportions in Socotra, Abd al Kuri and in the continent. We then measured the overlap between continental and island disparities by calculating the ratios between the disparities of Socotra and the continent, and between Abd al Kuri and the continent. These ratios were then compared with a null model consisting of 10,000 simulations in which body size and head proportions were stochastically simulated according to a Brownian motion model. Simulations were based on an empirical estimate of the rate parameter for body size, and on the estimated evolutionary variance-covariance matrix for head proportions.

By comparing the empirical ratios with the simulated ratios according to the stochastic model, we assessed whether island disparities significantly departed from the mean continental disparity. This analysis was performed on the summary tree, and it was also replicated for each of the 1,500 trees in order to incorporate the phylogenetic uncertainty into our empirical and simulated disparity ratios. All the analyses of this section were performed using the “ape^62^” and “geiger^61^” packages in R.

### Testing for differences in rates of phenotypic diversification

We used three different approaches to test whether insular and continental groups differed in their tempos of phenotypic evolution.

### OUwie

For the summary tree, we fitted BMS, BM1 and OU1 (described in Results) for each trait on each of the 1,000 ancestral state stochastic reconstructions of island-continent transitions described before (which reflected the uncertainty of the assignation of categories across the tree). For the set of 1,500 trees, the three models were fitted on each of the stochastic reconstructions conducted on each of the trees (which reflected the uncertainty in both the assignation of categories across the tree plus phylogenetic uncertainty). The three models were evaluated by comparing the AICc distributions and mean values. All analyses were conducted by the function “OUwie” in the R package “OUwie^63^,^64^” (more complex models available in this package were not fitted given our small dataset^64^).

In the case of body size, the only trait for which a multi-rate model (BMS) was most supported than single rate models, we also examined the support of this model against the single rate model (BM1) through parametric bootstrapping, using the protocol described in ref. 65. We performed this procedure using 1,000 bootstrap replicates, for each simmap reconstruction in the summary tree and for each reconstruction performed on each of the 1,500 trees of the posterior distribution. For each reconstruction and/or tree, we calculated the difference of log likelihoods between BM1 and BMS (the test statistic of the method) and computed the probability of having a difference at least as large as those computed in simulated datasets generated under BM1, as well as the statistic power of the test at 95% of confidence. We used the functions “brownie.lite” and “sim.rates” of the package “Phytools^58^”, in order to fit models and simulate according to given rate parameters, respectively.

### Auteur

Within the R package “auteur^66^” we conducted a reversible-jump Markov Chain Monte Carlo sampling to estimate the rates of evolution of all traits examined in this study (body size and each of the measurements of head variation). In this approach, rates were estimated along the branches of our summary tree with no prior assumption that evolutionary rates had changed at specific points in the phylogeny^66^. The analysis consisted in three independent chains that ran for 20*10^6^ generations each, with a sampling interval of 3,000 generations. The posterior estimates of these three runs were subsequently pooled with the first 10% of generations excluded as “burnin”. These analyses allowed us to estimate (and visualize) the posterior rates of evolution for each trait along branches. For each trait, we also compared the support of multiple versus single rate models by means of Bayes factors (BF). For body size (the only trait that presented rate heterogeneity; see Results), we replicated the analysis for each of the 1,500 trees to ensure that this rate heterogeneity could still be detected when topological and branch length uncertainties were incorporated into the analysis. In this case, for each of the 1,500 trees, we ran a single chain of 2*10^6^ generations with a sample interval set at 1,000 generations. For each tree we compared the support of multiple- versus single-rate models by means of BF and we also detected the clade (or lineages) associated with the rate shift that presented the highest computed posterior probability.

### Independent contrasts

To investigate how the heterogeneity in the rates of body size evolution was structured along time, we computed the absolute values of the standardized independent contrasts (another proxy for Brownian rates of evolution^67^,^68^) and plotted them against the height of the node that produced them. To assess whether the computed contrasts were in the range of the expected values assuming a single Brownian rate operating across the tree, we computed 10,000 simulated datasets generated by Brownian motion using an empirical estimate of the rate parameter. We then computed all independent contrasts for each simulated dataset and plotted the 95% of the contrast variation.

## Additional Information

**How to cite this article**: Garcia-Porta, J. *et al.* Testing the island effect on phenotypic diversification: insights from the *Hemidactylus* geckos of the Socotra Archipelago. *Sci. Rep.* **6**, 23729; doi: 10.1038/srep23729 (2016).

## Supplementary Material

Supplementary Information

## Figures and Tables

**Figure 1:**
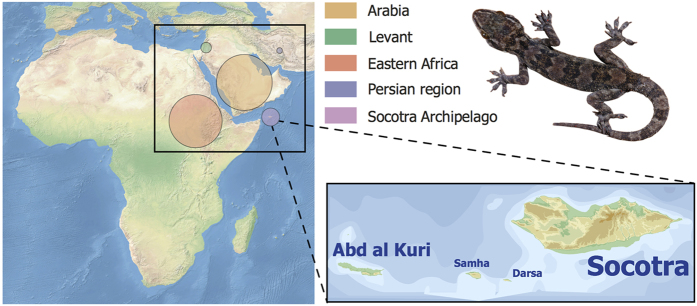
Map showing the geographic limits of this study. The diameters of the circles are proportional to the species richness of the arid clade of *Hemidactylus* within each geographic region. The map on the left was generated using ArcGIS 10.3 in WGS84 projection based on a map from Natural Earth (http://www.naturalearthdata.com/). The map on the right was made in Inkscape 0.91 (https://inkscape.org), with the boundaries and topography based on the Shuttle Radar Topography Mission data (http://www2.jpl.nasa.gov/srtm/) (designed by Oona Räisänen). The gecko shown in the figure is *Hemidactylus granti* (picture by Roberto Sindaco).
