# Supplementary material for: Testing the island effect on phenotypic diversification: insights from the Hemidactylus geckos of the Socotra Archipelago
Source: Sci Rep. 2016 Apr 13;6:23729. doi: 10.1038/srep23729 (PMC4829864; doi:10.1038/srep23729)
Supplement: Supplementary Information [file srep23729-s1.pdf]

Supplementary Material for:

TESTING THE ISLAND EFFECT ON PHENOTYPIC DIVERSIFICATION: INSIGHTS FROM THE HEMIDACTYLUS GECKOS OF THE SOCOTRA ARCHIPELAGO

Joan Garcia-Porta*, Jiří Šmíd, Daniel Sol, Mauro Fasola and Salvador Carranza

*To whom correspondence should be addressed

BEAST ANALYSIS

The phylogenetic analyses were conducted by means of the package BEAST v1.6.2 [1]. The prior for the distribution of branching times was based on a birth-death process. The variation of nucleotide substitution rates across the tree was assumed to be non-autocorrelated and log-normally distributed. The nucleotide substitution models (see above) were applied to the six partitions and the global substitution rate was set to one. This produced branch lengths expressed in units of substitutions per site (relative time).

As outgroups, we used the sequences of the same genes for six species known to be outside the arid clade (*H. mabouia*, *H. platycephalus*, *H. smithi*, *H. flaviviridis*, *H. angulatus* and *H. ruspolii*) [2].

We ran two independent Markov Chain Monte Carlo (MCMC) analyses for 50×10^6 generations, with parameters and trees sampled every 5,000 generations. These two independent runs converged on very similar posterior estimates and were combined using LogCombiner v1.6.2 (included in the package BEAST) after excluding the first 10% generations in each MCMC

chain. Tracer v.1.5 [1] was used to confirm convergence and good mixing of each of the MCMC chains (with all runs with ESS values > 200).

We calculated the summary tree as the maximum clade credibility tree with median node heights using TreeAnnotator v1.6.2 (also included in BEAST package), setting the posterior probability limit at 0.5. To incorporate the phylogenetic uncertainty into our comparative analyses (see below), we resampled the posterior distribution of trees resulting from our BEAST analysis to obtain a sample of 1,500 trees that varied in topology and branch lengths.

REFERENCES

1. Drummond, A. J. & Rambaut, A. BEAST: Bayesian evolutionary analysis by sampling trees. *BMC Evol. Biol.* **7,** 214 (2007).

2. Šmíd, J. *et al.* Out of Arabia: A complex biogeographic history of multiple vicariance and dispersal events in the gecko genus Hemidactylus (Reptilia: Gekkonidae). *PLoS One* **8,** e64018 (2013).

SUPPLEMENTARY FIGURES AND TABLES

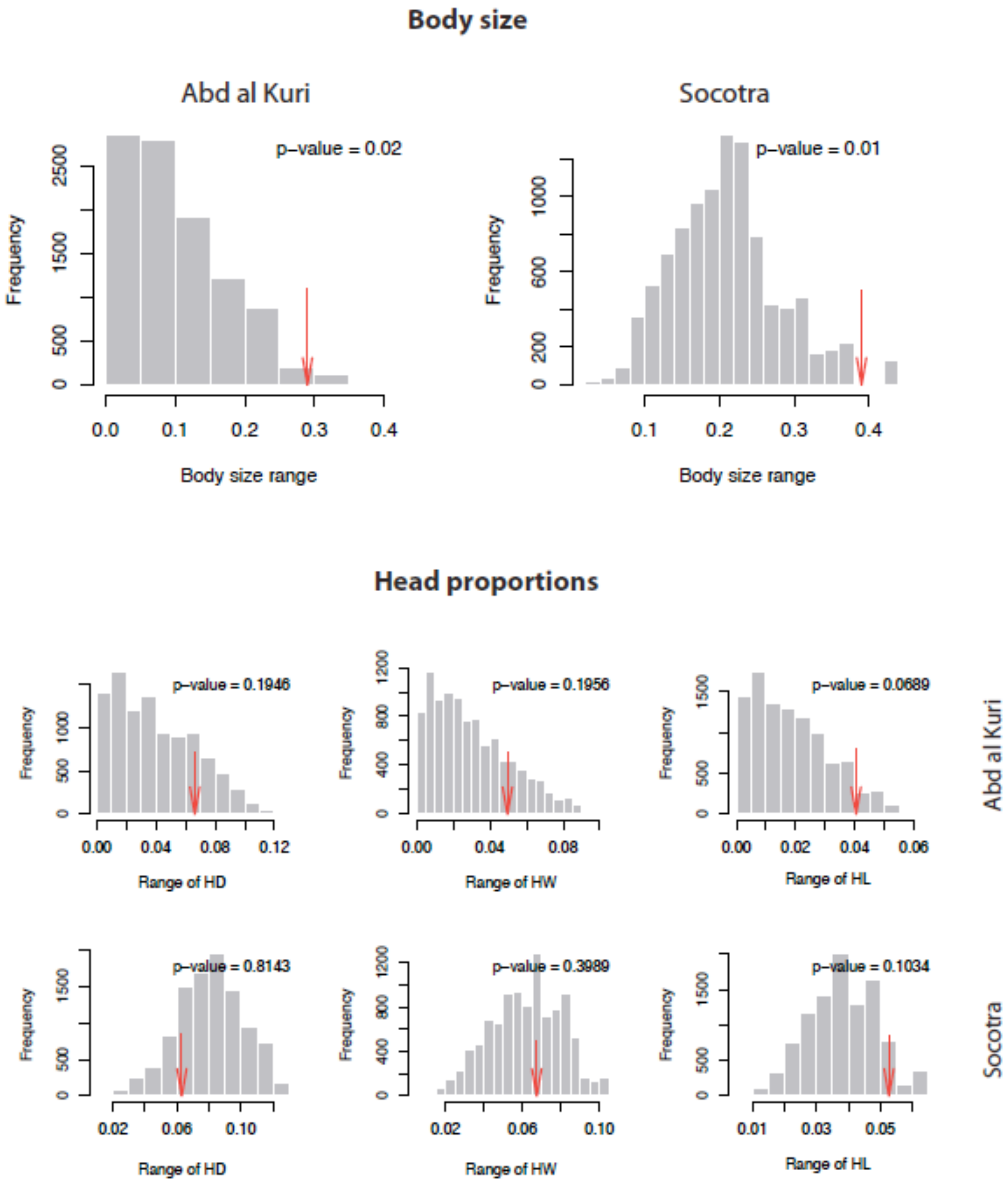

Figure S1. Distribution of the range variation (maximum – minimum value) in body size (SVL: snout-vent length) and in head proportions (HD: head depth, HW: head width, HL: head length) obtained by resampling 10,000 times the pool of continental species. Each sample simulated insular communities with two species in Abd al Kuri and five species in Socotra. Empirical values are indicated by red arrows. Also shown is the *p*-value associated to the empirical value given each distribution.

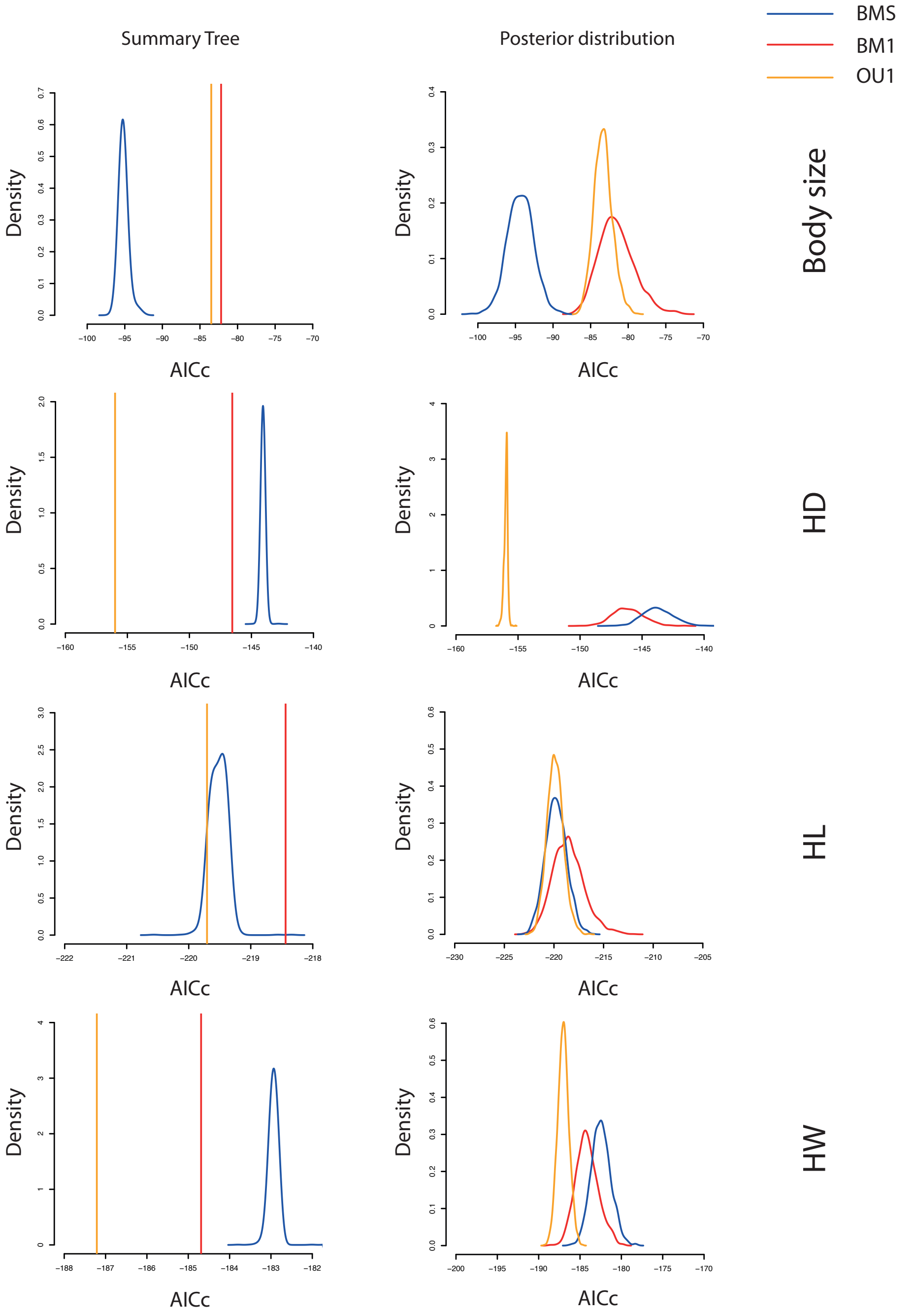

Figure S2. Distributions of the AICc values computed for model BMS (assuming rate heterogeneity), BM1 (assuming a single rate across the tree) and model OU1 (assuming a single rate plus one global optimum) for each of the measured traits. These curves have been

computed on the summary tree (left) and on the set of 1,500 trees obtained from the posterior distribution (right). HD: head depth, HW: head width, HL: head length.

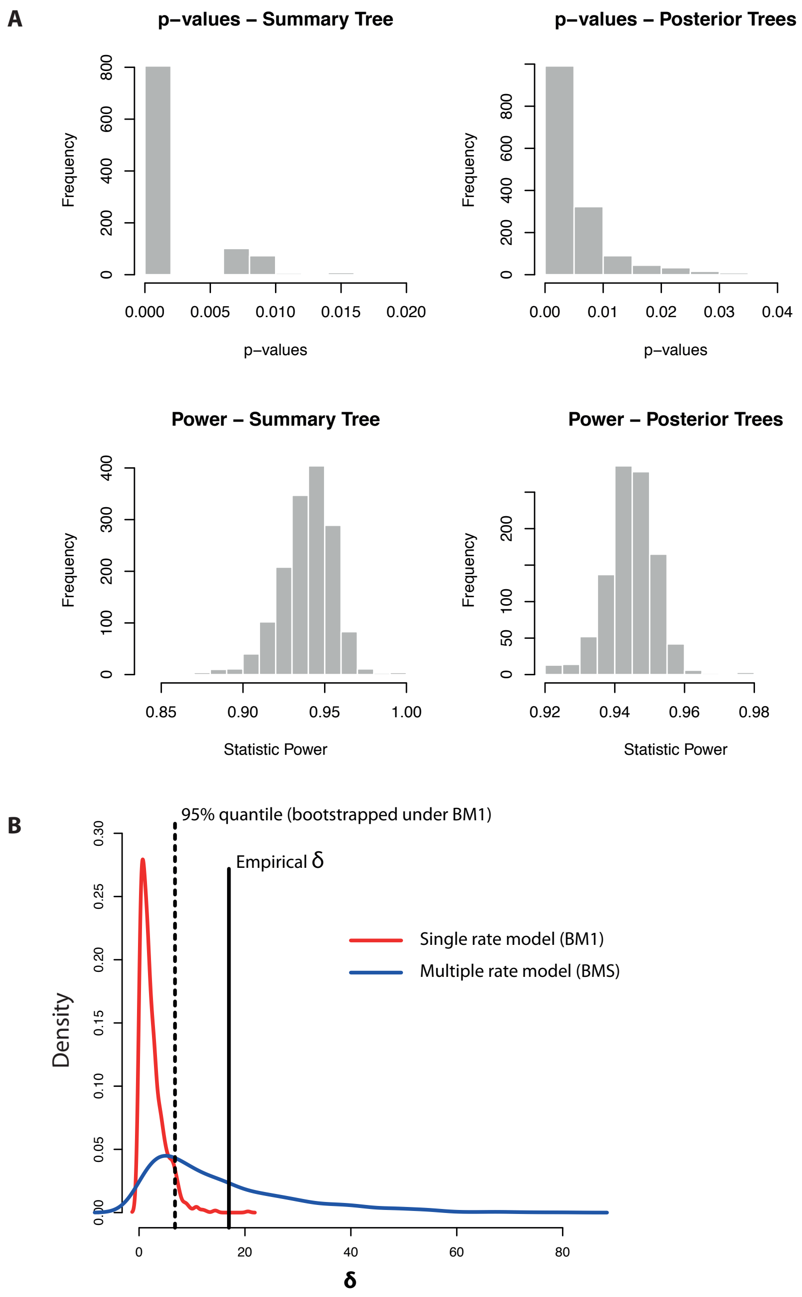

Figure S3. (A) Distributions of the *p*-values and estimates of statistic power, obtained by parametric bootstrapping using the method proposed in Boettiger et al., (2011). These histograms have been computed on the 1,000 reconstructions of the summary tree (left) and on

each of the reconstructions performed on the set of 1,500 trees obtained from the posterior distribution (right). (B) Example of the distributions of the differences of log likelihoods between the models BM1 and BMS (δ), obtained by bootstrapping under model BM1 (red) and model BMS (blue) (based on 1,000 replicates in one of the reconstructions of the summary tree). The dashed line indicates the position of the 95% quantile of the curve generated under model BM1. The solid line indicates the position of the observed δ of this particular reconstruction.

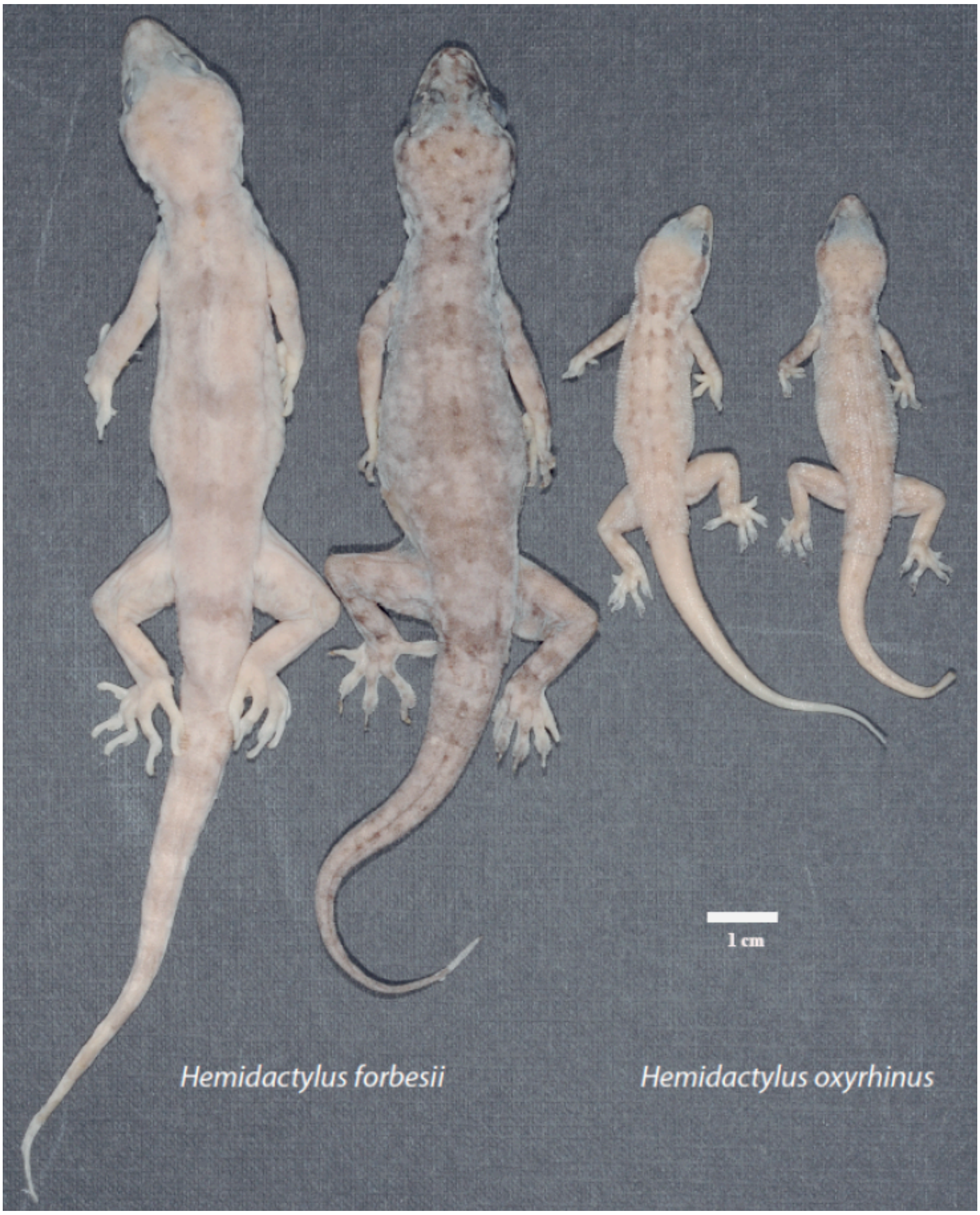

Figure S4. Difference in size between the two sister species in Abd al Kuri: *Hemidactylus forbesii* (left) and *H. oxyrhinus* (right). For both species, the two specimens depicted are male (left) and female (right). Picture by Salvador Carranza.

TABLES

Table S1. List of the species included in the phylogenetic analyses with their GenBank accession numbers for each of the genes used.

SPECIES	CODE	VOUCHER	COUNTRY	LOCALITY	12S	cytb	cmos	mc1r	rag2	rag1
H. adensis	BJ10	NHM-BS N41907	Yemen	Shaikh Othman	KP238276	KP238262	KP238253	KP238246	KP238233	KP238239
H. albopunctatus	JS207	TMHC 2012.07.087	Ethiopia	42 km SE of Jijiga	KC818657	KC818794	KC818745	KC818901	KC819017	KC818952
H. alkiyumii	JS2	NMP6V 74799/1	Yemen	3 km E Hauf	JQ957090	KC818795	JQ957123	JQ957244	JQ957401	KC818953
H. angulatus	JS123	NMP6V 74845/2	Ethiopia	Arba Minch	KC818659	KC818807	KC818747	KC818903	KC819018	KC818956
H. awashensis	JS212	NMP6V 74977	Ethiopia	2 km N of Metehara	KC818723	KC818873	KC818786	KC818939	KC819058	KC818998
H. barbierii	JS271	MSNPV-CR849	Kenya	Marsabit district, lagga Daudi	KU955711	KU955717	KU955714	KU955723	KU955740	KU955727
H. barodanus	JS211	TMHC 2012.07.091	Somalia	Laas Geel	KC818669	KC818814	KC818750	KU955724	KU955741	KU955728
H. citernii	JS203	TMHC 2012.07.095	Somalia	Laas Geel	KC818670	KC818815	KC818751	KC818907	KC819022	KC818958
H. dawudazraqi	Hd52	NMP6V 74134/1	Jordan	Azraq	KC818671	HQ833753	JQ957161	JQ957299	JQ957423	KC818960
H. dracaenacolus	IBES2604	unvouchered	Yemen	Tahr Diksam, Socotra Island	JQ982781	JQ982889	KC818752	JQ982643	KC819023	KU955729
H. festivus	JS1	NMP6V 74812	Yemen	Wadi Hadramauth	JQ957096	KC818816	JQ957125	KC818909	JQ957409	KC818961
H. flaviviridis	JS111	unvouchered	Pakistan	Okara	KC818676	KC818822	JQ957126	JQ957253	KC819026	KC818965
H. forbesii	IBE5432	unvouchered	Yemen	Al Alyah, Abd al Kuri Island	JQ982786	JQ982894	KC818756	JQ982647	JQ957425	KU955730
H. foudaii	JS151	NMP6V 74808	Sudan	15 km SE of Atbara	KC818677	KU955720	KC818757	KC818912	KC819029	KC818968
H. funaiolii	JS196	NMP6V 74452	Kenya	Hurri Hills	KC818678	KC818827	KC818758	KC818913	KC819030	KC818969
H. granchii	JS214	TMHC 2012.07.088	Somalia	40 km NE of Hargeisa	KC818679	KC818828	JQ957138	KC818914	KC819031	KC818970
H. granosus	HSA3	IBES10065	Saudi Arabia	Taif National Wildlife Research Center	KU955712	KU955718	KU955715	KU955725	KU955742	KU955731
H. granti	IBES5307	unvouchered	Yemen	Adho Dimellus, Socotra Island	JQ982788	JQ982902	KC818759	JQ982652	KC819023	KU955732
H. hajarensis	JS98	NMP6V 74860/1	Oman	Muqal	JQ957101	KC818831	JQ957128	KC818915	JQ957419	KC818972
H. homoeolepis	S3399	Unvouchered	Yemen	Hadibo, Socotra Island	JQ957059	JQ957193	JQ957132	JQ957263	JQ957422	KJ189938
H. inexpectatus	S2166	BM2008.711	Oman	2.5 km SE Ar Rumayliyah	JQ957067	JQ957207	JQ957140	JQ957275	JQ957426	KU955733
H. inintellectus	IBES5068	unvouchered	Yemen	Wadi Ayahft, Socotra Island	JQ982802	JQ982922	KC818766	JQ982673	KC819035	KU955734
H. isolepis	JS202	NMP 74447	Somalia	8 km S of Borama	KC818680	KC818835	KC818769	KC818917	KC819036	KC818974
H. jumailiae	JS53	NMP6V 74818/1	Yemen	25 km NW of Al Bayda	KC818682	KC818837	KC818770	KC818918	KC819038	KC818975
H. lavadeserticus	Hd70	NMP6V 74049/2	Syria	Ar´Raqiyeh	KC818683	HQ833743	JQ957161	KC818919	KC819039	KC818976
H. lemurinus	JS11	NMP6V 74801/2	Yemen	Damqawt	JQ957113	KC818839	JQ957137	KC818920	JQ957423	KC818977
H. luqueorum	AO46	BM2005.1660	Oman	Sayq, Jebel Akhdar	JQ957069	JQ957212	JQ957143	JQ957279	JQ957429	-
H. mabouia	JS173	NMP6V 74804	Uganda	Mpanga forest	KC818685	KC818840	KC818771	KC818922	KC819040	KC818979
H. macropholis	JS226	TMHC2012.01.032	Ethiopia	10 km N of Degeh Bur	KU955713	KU955719	KU955716	KU955726	KU955743	KU955735
H. mandebensis	JS27	NMP6V 74970	Yemen	Jabal Sabir	KC818732	KC818883	KC818790	KP238244	JQ957409	KP238241
H. masirahensis	S7710	IBES7710	Oman	Wadi Maahdi, Masirah island	JQ957063	JQ957204	JQ957139	JQ957272	JQ957425	KU955736
H. mindiae	Hd22	NMP6V 72323/2	Jordan	Jabal Ghazali	KC818686	HQ833747	JQ957128	-	JQ957423	KC818980
H. minutus	S7966	IBES7966	Oman	14.5 km NE of Sharbthat	JQ957057	JQ957197	JQ957136	JQ957262	JQ957422	KJ189962
H. modestus	CAS198934	CAS198934	Kenya	Kijiado District, Rift Valley Province	DQ120386	DQ120215	JQ957149	JQ957283	JQ957432	-
H. ophiolepis	JS215	TMHC 2012.07.094	Ethiopia	15 km NE of Dire Dawa	KC818689	KC818841	KC818772	KC818923	KC819042	KC818982
H. oxyrhinus	IBE5394	unvouchered	Yemen	Bir Al Aguz ,Abd al Kuri Island	JQ982810	JQ982935	KC818773	JQ982677	JQ957425	KU955737
H. paucituberculatus	S7646	IBES7646	Oman	Khor Sauli	JQ957072	JQ957217	JQ957150	JQ957287	JQ957425	KU955738
H. persicus	JS103	NMP6V 74807/1	Iran	Booreki	KC818691	KC818842	KC818775	KC818924	KC819044	KC818983
H. platycephalus	JS169	NMP6V 74864/2	Kenya	Ngurunit	KC818693	KC818845	KC818776	KC818926	KC819045	KC818985
H. pumilio	IBES5021	unvouchered	Yemen	Steroh, Socotra Island	JQ982817	JQ982948	KC818777	JQ982693	KC819047	KU955739
H. robustus	JS101	unvouchered	Somalia	Berbera	KC818696	KC818847	KC818778	KC818927	JQ957409	KC818987
H. ruspolii	JS177	NMP6V 74871/3	Kenya	Kalacha	KC818706	KC818860	KC818782	KC818931	KC819052	-
H. saba	BJ28	NHM-BS N41913	Yemen	Marib	KF647567	KF647579	KF647573	KF647586	KF647605	-
H. shihraensis	JS16	NMP6V 74816 Yemen 11 km N of Mukalla	Yemen	11 km N of Mukalla	KC818710	KU955721	KC818783	KC818932	KC819053/KC818990	KC819053 KC818990
H. sinaitus	JS146	NMP6V 74809/1	Sudan	Wad Ben Naga	KC818712	KC818866	JQ957164	KC818935	JQ957446	KC818993
H. smithi	JS208	TMHC 2012.07.086	Somalia	30 km N of Shiikh	KC818715	KC818870	KC818785	KC818936	KC819055	KC818996
H. sp. 10	JS181	unvouchered	Kenya	Gus	KC818716	KC818871	KC818769	KC818937	KC819056	KC818997
H. sp. 9	JS216	unvouchered	Ethiopia	10 km E of Yidi	KC818736	KC818887	KC818792	KC818945	KC819064	KC819004
H. squamulatus	JS183	NMP6V 74972	Kenya	Isiolo	KC818739	KU955722	JQ957149	KC818946	KC819065	KC819005
H. turcicus	Hd2	unvouchered	Israel	Almagor	KC818740	HQ833741	KC818793	KC818948	JQ957423	KC819007
H. ulii	JS17	NMP6V 74831/1	Yemen	Al Hadr	KC818725	KC818874	KC818787	KC818940	KC819059	KC818999
H. montanus	JS52	NMP6V 74803	Yemen	5 km NE of Hajjah	KC818688	KC818900	KC818750	KC818951	KC819070	KC819016
H. pauciporosus	CAS227511	CAS227511	Somalia	11 km SE of Bosasso	DQ120379	DQ120208	JQ957138	JQ957270	JQ957424	-
H. yerburii	JS40	NMP6V 74824/1	Yemen	3 km S of Najd an Nashamah	JQ957086	KC818894	JQ957167	JQ957306	JQ957447	KC819013

Table S2. Measurements of all species of *Hemidactylus* included in the morphological analyses. Institutions: BMNH = British Museum of Natural History, London, UK; CAS = California Academy of Sciences, California, USA; IBE = Institute of Evolutionary Biology, Barcelona, Spain; MCCI = MCCI—Museo Civico di Storia Naturale di Carmagnola, Turin, Italy; MSNPV = Museo di Storia Naturale of the University of Pavia, Italy; MSNG = Museo Civico di Storia Naturale 'Giacomo Doria', Genova, Italy; MSNM = Museo Civico di Storia Naturale, Milano, Italy; MZUF = MZUF—Museo di Storia Naturale del l'Università di Firenze "La Specola", Firenze, Italy; NHM-BS = Naturhistorischesn Museum Braunschweig, Germany; NMP = National Museum in Prague, Prague, Czech Republic; ONHM = Oman Natural History Museum, Muscat, Oman; TMHC = Tomas Mazuch Herpetological Collection, Czech Republic. The abbreviations of measurements are: Body size = SVL, head length = HL, head width = HW, head height = HD.

SPECIES	INSTITUTION	VOUCHER CODE	COUNTRY	SVL (mm)	HL (mm)	HW (mm)	HD (mm)
H. adensis	NHM-BS	NHM-BS N41907	Yemen	29.91	7.95	5.88	3.23
H. adensis	NHM-BS	NHM-BS N41905	Yemen	30.11	8.32	6.06	3.34
H. adensis	NHM-BS	NHM-BS N41902	Yemen	34.33	8.78	6.43	3.56
H. adensis	NMP	NMP6V 74837	Yemen	41.84	10.24	7.26	4.31
H. adensis	NHM-BS	NHM-BS N41904	Yemen	37.94	10.30	7.84	4.33
H. adensis	NHM-BS	NHM-BS N41903	Yemen	40.64	10.33	7.81	4.32
H. albopunctatus	TMHC	TMHC 2012.07.087	Ethiopia	55.12	?	?	?
H. alkiyumii	NMP	NMP6V 74838/1	Oman	48.32	12.41	9.29	4.95
H. alkiyumii	BMNH	BMNH1977.973	Oman	46.70	12.60	10.30	6.50
H. alkiyumii	NMP	NMP6V 74799/9	Oman	48.67	13.06	9.91	5.24
H. alkiyumii	NMP	NMP6V 74838/4	Oman	50.38	13.47	9.80	6.44
H. alkiyumii	NMP	NMP6V 74840/2	Oman	47.47	13.49	9.25	5.54
H. alkiyumii	NMP	NMP6V 74840/1	Oman	48.17	13.58	11.15	5.66
H. alkiyumii	IBE	IBES7192	Oman	46.50	13.70	9.30	5.60
H. alkiyumii	NMP	NMP6V 74844/2	Oman	51.42	13.88	10.45	5.91
H. alkiyumii	CAS	CAS227519	Oman	49.81	14.09	10.68	6.47
H. alkiyumii	ONHM	ONHM3707P	Oman	49.50	14.10	10.60	6.60
H. alkiyumii	NMP	NMP6V 74840/3	Oman	52.70	14.18	11.42	6.26
H. alkiyumii	IBE	IBES7453	Oman	53.00	14.70	10.80	6.30
H. alkiyumii	BMNH	BMNH1977.965	Oman	55.00	14.70	11.80	6.90
H. alkiyumii	NMP	NMP6V 74838/2	Oman	56.10	14.76	11.88	6.77

H. alkiyumii	BMNH	BMNH1977.959	Oman	53.30	14.80	11.00	5.80
H. alkiyumii	NMP	NMP6V 74839/4	Oman	56.15	14.95	12.15	7.17
H. alkiyumii	NMP	NMP6V 74844/1	Oman	56.65	14.95	12.49	7.04
H. alkiyumii	IBE	IBES7897	Oman	55.30	15.00	11.50	5.90
H. alkiyumii	NMP	NMP6V 74838/3	Oman	58.67	15.06	12.04	7.08
H. alkiyumii	BMNH	BMNH1977.958	Oman	61.60	15.10	11.90	7.80
H. alkiyumii	IBE	IBES7053	Oman	55.00	15.20	11.60	7.10
H. alkiyumii	IBE	IBES8080P	Oman	53.20	15.30	11.00	6.70
H. alkiyumii	BMNH	BMNH1977.966	Oman	53.30	15.40	11.60	6.60
H. alkiyumii	BMNH	BMNH2005.1662H	Oman	56.40	15.40	11.50	7.50
H. alkiyumii	IBE	IBES7891	Oman	56.80	15.40	11.50	7.00
H. alkiyumii	BMNH	BMNH1977.964	Oman	58.60	15.50	12.70	7.40
H. alkiyumii	NMP	NMP6V 74844/3	Oman	59.24	15.63	12.65	7.24
H. alkiyumii	IBE	IBES8079P	Oman	58.00	15.70	12.10	6.70
H. alkiyumii	IBE	IBES7858	Oman	57.00	16.00	11.10	7.00
H. alkiyumii	IBE	IBES7441	Oman	57.30	16.10	12.10	8.30
H. alkiyumii	NMP	NMP6V 74839/1	Oman	60.77	16.12	12.67	7.73
H. alkiyumii	NMP	NMP6V 74842/2	Oman	60.84	16.37	13.19	6.94
H. alkiyumii	BMNH	BMNH1977.956	Oman	60.10	16.60	12.30	8.00
H. alkiyumii	BMNH	BMNH1977.963	Oman	65.50	16.70	13.70	8.90
H. alkiyumii	IBE	IBES7888	Oman	62.50	16.80	13.30	7.80
H. alkiyumii	IBE	IBES7879	Oman	62.50	17.00	13.20	7.80
H. alkiyumii	BMNH	BMNH1976.1409	Oman	64.20	17.00	13.50	8.70
H. alkiyumii	BMNH	BMNH1977.957	Oman	61.00	17.20	13.10	8.00
H. alkiyumii	IBE	IBES8078P	Oman	62.30	17.30	13.20	8.30
H. alkiyumii	IBE	IBES7101	Oman	62.40	17.30	12.80	7.90
H. alkiyumii	IBE	IBES7837	Oman	67.50	17.70	14.70	8.90
H. alkiyumii	NMP	NMP6V 74844/4	Oman	69.42	18.06	13.93	8.49
H. alkiyumii	IBE	IBES7397	Oman	66.10	18.10	14.60	9.10
H. alkiyumii	NMP	NMP6V 74839/2	Oman	69.79	18.15	14.86	8.85
H. alkiyumii	BMNH	BMNH1977.972	Oman	74.50	18.40	15.60	9.30
H. alkiyumii	NMP	NMP6V 74839/3	Oman	71.77	18.41	15.84	9.02
H. alkiyumii	NMP	NMP6V 74841	Oman	68.86	18.49	14.80	8.80
H. alkiyumii	BMNH	BMNH2005.1663P	Oman	68.60	18.70	14.50	9.30
H. alkiyumii	NMP	NMP6V 74799/11	Yemen	42.56	11.97	8.96	5.17
H. alkiyumii	NMP	NMP6V 74799/2	Yemen	52.08	13.98	10.93	6.55
H. alkiyumii	NMP	NMP6V 74799/1	Yemen	51.03	14.04	10.43	6.33
H. alkiyumii	NMP	NMP6V 74799/3	Yemen	54.57	14.61	11.06	6.05
H. alkiyumii	NMP	NMP6V 74799/10	Yemen	56.88	15.31	11.06	6.84
H. alkiyumii	NMP	NMP6V 74800	Yemen	59.31	15.92	12.61	7.50
H. alkiyumii	NMP	NMP6V 74799/7	Yemen	64.83	17.02	12.44	7.93
H. alkiyumii	NMP	NMP6V 74799/5	Yemen	63.55	17.26	13.00	7.31
H. alkiyumii	NMP	NMP6V 74799/6	Yemen	63.46	17.47	13.48	8.39
H. alkiyumii	NMP	NMP6V 74799/8	Yemen	64.61	17.58	13.19	8.84
H. alkiyumii	NMP	NMP6V 74799/4	Yemen	66.71	17.63	13.78	8.15

H. awashensis	MSNG	MSNG31405	Eritrea	37.74	11.20	8.20	4.85
H. awashensis	NMP	NMP6V 74977	Ethiopia	52.44	13.59	10.62	5.62
H. awashensis	MZUF	MZUF22202	Ethiopia	54.16	14.69	11.78	6.08
H. barbierii	MSNPV	MSNPV-CR849	Kenya	40.00	?	?	?
H. barodanus	TMHC	TMHC 2012.07.081	Ethiopia	46.40	12.55	9.62	5.17
H. barodanus	TMHC	TMHC2012.07.085	Ethiopia	59.79	14.77	11.42	5.94
H. barodanus	TMHC	TMHC2012.07.082	Ethiopia	59.81	14.94	12.47	6.91
H. barodanus	TMHC	TMHC2012.07.083	Ethiopia	62.60	16.22	13.40	6.81
H. citernii	TMHC	TMHC2012.07.099	Somalia	37.80	9.57	7.39	3.87
H. dawudazraqi	NMP	NMP6V 74135/7	Jordan	31.29	8.32	6.52	3.77
H. dawudazraqi	NMP	NMP6V 74135/6	Jordan	33.17	8.78	6.67	4.15
H. dawudazraqi	NMP	NMP6V 74136/7	Jordan	36.23	10.74	7.69	4.54
H. dawudazraqi	NMP	NMP6V 72130/3	Jordan	41.40	10.81	9.07	5.84
H. dawudazraqi	NMP	NMP6V 74134/1	Jordan	46.38	10.91	9.47	5.95
H. dawudazraqi	NMP	NMP6V 72740/2	Jordan	49.87	11.99	10.00	6.02
H. dawudazraqi	NMP	NMP6V 72130/2	Jordan	46.38	12.28	9.71	5.67
H. dawudazraqi	NMP	NMP6V 72130/1	Jordan	45.99	12.38	11.23	6.98
H. dawudazraqi	NMP	NMP6V 72740/1	Jordan	48.66	12.57	10.48	6.14
H. dawudazraqi	NMP	NMP6V 74137	Jordan	50.04	14.00	10.70	5.71
H. dawudazraqi	NMP	NMP6V 74136/1	Jordan	52.19	14.23	11.03	6.37
H. dawudazraqi	NMP	NMP6V 70457	Syria	35.64	9.80	7.78	4.71
H. dracaenacolus	MCCI	R1502-2	Yemen (Socotra Archipelago)	52.80	15.10	11.50	7.20
H. dracaenacolus	MSNPV	Y35	Yemen (Socotra Archipelago)	66.00	18.00	14.40	8.40
H. dracaenacolus	MCCI	R1578-1	Yemen (Socotra Archipelago)	64.90	18.10	14.00	7.20
H. dracaenacolus	MCCI	R1578-2	Yemen (Socotra Archipelago)	61.40	18.20	14.10	7.80
H. dracaenacolus	MCCI	R1502-1	Yemen (Socotra Archipelago)	69.20	19.50	16.20	10.00
H. festivus	BMNH	BMNH1977.981P	Oman	36.00	10.30	7.10	4.40
H. festivus	IBE	IBES7419P	Oman	40.60	11.60	8.30	4.90
H. festivus	NMP	NMP6V 74854/4	Oman	45.02	11.74	9.33	5.63
H. festivus	BMNH	BMNH1977.979P	Oman	43.70	11.80	8.90	5.30
H. festivus	NMP	NMP6V 74854/3	Oman	44.64	12.29	9.30	5.56
H. festivus	IBE	IBES8063	Oman	43.10	12.80	9.00	4.80
H. festivus	BMNH	BMNH1977.978P	Oman	48.70	13.00	9.60	5.90
H. festivus	BMNH	BMNH1977.977H	Oman	49.00	13.10	10.00	6.90
H. festivus	BMNH	BMNH1977.975	Oman	49.00	13.30	9.90	6.20
H. festivus	NMP	NMP6V 74854/1	Oman	50.17	13.45	11.20	6.52
H. festivus	BMNH	BMNH1983.706	Oman	51.40	13.50	10.90	7.30
H. festivus	NMP	NMP6V 74854/2	Oman	52.58	13.89	10.95	6.84
H. festivus	BMNH	BMNH1977.976P	Oman	53.30	14.00	10.20	6.10
H. festivus	ONHM	ONHM3708P	Oman	50.10	14.40	9.60	5.60
H. festivus	IBE	IBES8062P	Oman	51.50	14.50	10.50	6.90
H. festivus	IBE	IBES7616	Oman	53.10	14.50	9.20	6.10
H. festivus	IBE	IBES7899	Oman	52.60	14.60	10.20	6.70
H. festivus	IBE	IBES7159P	Oman	52.50	14.70	10.40	6.70
H. festivus	IBE	IBES7605P	Oman	53.60	15.80	10.50	6.20

H. festivus	NMP	NMP6V 74811/2	Yemen	38.68	10.54	7.98	5.05
H. festivus	NMP	NMP6V 74812	Yemen	42.05	11.51	8.43	5.12
H. festivus	NMP	NMP6V 74811/1	Yemen	45.02	12.62	9.22	5.76
H. forbesii	MCCI	R1579a	Yemen (Socotra Archipelago)	81.00	18.60	15.50	10.10
H. forbesii	MCCI	R1579b	Yemen (Socotra Archipelago)	74.20	19.40	14.50	8.90
H. forbesii	MCCI	R1579b	Yemen (Socotra Archipelago)	74.90	19.60	15.00	8.80
H. forbesii	MCCI	R1579a	Yemen (Socotra Archipelago)	78.70	20.90	15.40	9.60
H. forbesii	MCCI	R1579b	Yemen (Socotra Archipelago)	85.40	21.40	16.80	10.40
H. forbesii	MCCI	R1579a	Yemen (Socotra Archipelago)	85.40	22.00	16.60	10.90
H. forbesii	MCCI	R1579a	Yemen (Socotra Archipelago)	87.00	22.50	16.80	10.70
H. forbesii	MCCI	R1579b	Yemen (Socotra Archipelago)	92.20	22.50	17.10	9.90
H. foudaii	NMP	NMP6V 74808	Sudan	47.03	12.52	9.90	5.57
H. funaiolii	Lanza (1978)		Kenya	32.00	?	?	?
H. funaiolii	Lanza (1978)		Kenya	32.00	?	?	?
H. funaiolii	Lanza (1978)		Kenya	33.00	?	?	?
H. granchii	TMHC	TMHC 2012.07.088	Somalia	50.03	13.34	10.53	5.57
H. granosus	NMP	NMP6V 70163/3	Egypt	48.58	11.90	9.11	4.94
H. granosus	NMP	NMP6V 70163/1	Egypt	47.48	12.77	8.92	5.19
H. granosus	NMP	NMP6V 70163/2	Egypt	53.17	13.19	9.85	6.16
H. granosus	IBE	IBES10132	Saudi Arabia	39.27	9.68	7.83	3.82
H. granosus	IBE	IBES10062	Saudi Arabia	40.59	10.13	8.06	4.10
H. granosus	IBE	IBES10065	Saudi Arabia	47.24	10.70	9.09	3.91
H. granosus	IBE	IBES10284	Saudi Arabia	39.01	11.06	8.02	3.66
H. granosus	IBE	IBES10150	Saudi Arabia	44.12	11.73	10.13	4.01
H. granosus	IBE	IBES10344	Saudi Arabia	49.79	12.29	10.47	4.62
H. granosus	IBE	IBES10361	Saudi Arabia	44.37	12.39	9.10	4.40
H. granosus	IBE	IBES10213	Saudi Arabia	48.41	12.43	9.53	4.61
H. granosus	IBE	IBES10127	Saudi Arabia	53.25	12.74	9.66	5.24
H. granosus	IBE	IBES10351	Saudi Arabia	50.75	13.02	9.17	4.80
H. granosus	IBE	IBES10212	Saudi Arabia	52.89	13.04	9.21	4.65
H. granosus	IBE	IBES10318	Saudi Arabia	48.59	13.24	8.97	5.32
H. granosus	IBE	IBES10183	Saudi Arabia	49.94	13.62	10.62	5.83
H. granosus	IBE	IBES10363	Saudi Arabia	53.13	13.70	9.97	6.13
H. granti	MCCI	R1501-2008	Yemen (Socotra Archipelago)	62.70	16.00	12.80	7.50
H. granti	MCCI	R1501-2009	Yemen (Socotra Archipelago)	60.00	16.50	12.80	7.30
H. granti	MSNPV	TR22	Yemen (Socotra Archipelago)	58.60	16.90	12.20	7.40
H. granti	MCCI	R1606-1	Yemen (Socotra Archipelago)	58.60	17.10	12.00	6.50
H. granti	MCCI	R1606-2	Yemen (Socotra Archipelago)	70.10	19.30	14.20	8.00
H. hajarensis	BMNH	BMNH2008.708	Oman	40.00	12.20	8.20	5.30
H. hajarensis	BMNH	BMNH1976.1404	Oman	50.00	13.20	9.70	6.10
H. hajarensis	IBE	IBES8064	Oman	47.80	13.50	10.00	6.00
H. hajarensis	IBE	IBES1777	Oman	46.20	13.60	9.00	5.40
H. hajarensis	BMNH	BMNH2008.707	Oman	44.30	13.70	9.50	5.20
H. hajarensis	IBE	IBES7335P	Oman	47.10	13.70	9.30	5.60
H. hajarensis	BMNH	BMNH1977.35	Oman	57.40	13.70	11.70	7.60

H. hajarensis	IBE	IBES7336P	Oman	46.70	13.80	9.60	6.00
H. hajarensis	NMP	NMP6V 74860/2	Oman	52.22	14.11	10.81	6.30
H. hajarensis	BMNH	BMNH2008.706	Oman	48.70	14.20	9.90	5.80
H. hajarensis	BMNH	BMNH2008.701	Oman	52.00	14.40	10.60	6.20
H. hajarensis	NMP	NMP6V 74861	Oman	55.93	15.08	11.51	7.25
H. hajarensis	ONHM	ONHM3706P	Oman	52.80	15.20	10.60	6.10
H. hajarensis	IBE	IBES7184	Oman	57.50	15.50	11.40	5.80
H. hajarensis	BMNH	BMNH2008.714H	Oman	59.80	15.50	12.70	7.40
H. hajarensis	IBE	IBES7151	Oman	56.70	15.60	12.20	5.60
H. hajarensis	BMNH	BMNH2008.704	Oman	54.60	15.70	10.20	7.20
H. hajarensis	NMP	NMP6V 74862	Oman	56.16	15.80	11.83	7.97
H. hajarensis	BMNH	BMNH2005.1664	Oman	56.00	16.00	11.50	7.10
H. hajarensis	IBE	IBES7076	Oman	60.40	16.60	11.60	6.80
H. hajarensis	BMNH	BMNH2008.709	Oman	60.00	16.70	12.20	7.10
H. hajarensis	IBE	IBES7154	Oman	62.70	17.00	12.90	7.20
H. hajarensis	BMNH	BMNH2008.703	Oman	59.70	17.10	11.20	8.00
H. hajarensis	BMNH	BMNH2008.702	Oman	61.00	17.10	11.40	6.40
H. hajarensis	IBE	IBES7587	Oman	66.90	18.50	13.30	7.30
H. homoeolepis	BMNH	BMNH99.12.5..41	Yemen (Socotra Archipelago)	29.90	7.40	5.30	3.40
H. homoeolepis	BMNH	BMNH1953.1.7.86	Yemen (Socotra Archipelago)	37.80	7.50	6.50	4.30
H. homoeolepis	BMNH	BMHN1967.488	Yemen (Socotra Archipelago)	35.80	7.80	6.40	4.20
H. homoeolepis	BMNH	BMHN1967.489	Yemen (Socotra Archipelago)	30.50	7.90	5.50	3.60
H. homoeolepis	IBE	R1599 IBES5353	Yemen (Socotra Archipelago)	31.70	8.20	5.70	3.50
H. homoeolepis	BMNH	BMNH1953.1.7.83	Yemen (Socotra Archipelago)	34.60	8.40	6.20	4.10
H. homoeolepis	IBE	1090 IBES10573	Yemen (Socotra Archipelago)	29.20	8.50	5.70	4.00
H. homoeolepis	BMNH	BMNH1953.1.7.87	Yemen (Socotra Archipelago)	34.00	8.50	6.30	4.50
H. homoeolepis	IBE	R1599 IBES5306	Yemen (Socotra Archipelago)	34.70	8.60	6.00	3.60
H. homoeolepis	MSNPV	1173	Yemen (Socotra Archipelago)	31.10	8.70	5.80	3.60
H. homoeolepis	BMNH	BMNH 81.7.22.7	Yemen (Socotra Archipelago)	32.10	8.70	6.10	3.60
H. homoeolepis	BMNH	BMHN1967.485	Yemen (Socotra Archipelago)	39.10	8.70	7.60	4.40
H. homoeolepis	BMNH	BMHN1967.487	Yemen (Socotra Archipelago)	39.50	8.70	7.00	4.40
H. homoeolepis	BMNH	BMNH99.12.5.38	Yemen (Socotra Archipelago)	40.50	8.70	7.80	5.00
H. homoeolepis	IBE	R1598 IBES3562	Yemen (Socotra Archipelago)	32.30	8.90	6.50	3.80
H. homoeolepis	IBE	R1516 IBES3289	Yemen (Socotra Archipelago)	33.30	8.90	6.70	3.70
H. homoeolepis	IBE	1092 IBES10575	Yemen (Socotra Archipelago)	34.90	9.00	7.00	4.20
H. homoeolepis	BMNH	BMNH99.12.5..40	Yemen (Socotra Archipelago)	39.60	9.00	7.40	4.80
H. homoeolepis	BMNH	BMHN1967.490	Yemen (Socotra Archipelago)	39.70	9.00	6.50	4.40
H. homoeolepis	BMNH	BMNH99.12.5.42	Yemen (Socotra Archipelago)	40.00	9.00	7.20	4.80
H. homoeolepis	IBE	1059 IBES10576	Yemen (Socotra Archipelago)	34.00	9.10	5.80	3.80
H. homoeolepis	BMNH	BMHN1967.486	Yemen (Socotra Archipelago)	39.10	9.10	7.60	4.90
H. homoeolepis	IBE	R1598 IBES5189	Yemen (Socotra Archipelago)	34.00	9.20	6.30	3.80
H. homoeolepis	IBE	R1599 IBES5154	Yemen (Socotra Archipelago)	35.60	9.40	6.40	4.20
H. homoeolepis	BMNH	BMNH99.12.5.43	Yemen (Socotra Archipelago)	42.00	9.40	7.70	4.70
H. homoeolepis	IBE	1091 IBES10574	Yemen (Socotra Archipelago)	34.90	9.50	6.50	4.40
H. homoeolepis	IBE	1060 IBES10577	Yemen (Socotra Archipelago)	37.00	9.70	6.30	3.80

H. homoeolepis	MSNPV	1169	Yemen (Socotra Archipelago)	36.60	9.80	6.80	4.20
H. homoeolepis	IBE	1061 IBES10631	Yemen (Socotra Archipelago)	38.40	9.80	7.00	3.70
H. homoeolepis	IBE	R1517 IBESS10629	Yemen (Socotra Archipelago)	35.40	10.10	6.70	4.30
H. homoeolepis	IBE	R1598 IBES5305	Yemen (Socotra Archipelago)	38.80	10.20	6.70	4.10
H. homoeolepis	IBE	R1517 IBES3281	Yemen (Socotra Archipelago)	40.80	10.40	6.90	4.50
H. homoeolepis	BMNH	BMNH 81.7.22.6	Yemen (Socotra Archipelago)	38.50	10.50	7.50	4.80
H. homoeolepis	MSNPV	1054	Yemen (Socotra Archipelago)	39.00	10.60	7.90	4.80
H. homoeolepis	IBE	1062 IBES10630	Yemen (Socotra Archipelago)	38.10	10.70	7.70	4.20
H. homoeolepis	IBE	1062 IBES10579	Yemen (Socotra Archipelago)	40.00	10.70	6.80	4.30
H. homoeolepis	IBE	R1515 IBES3419	Yemen (Socotra Archipelago)	41.50	10.90	8.70	4.50
H. homoeolepis	IBE	R1504 IBES5326	Yemen (Socotra Archipelago)	43.20	11.20	8.00	3.60
H. homoeolepis	IBE	R1504 IBES3314	Yemen (Socotra Archipelago)	42.10	11.40	8.30	4.80
H. homoeolepis	IBE	R1518 IBES10687	Yemen (Socotra Archipelago)	43.70	11.60	7.80	4.60
H. homoeolepis	IBE	R1518 IBES10745	Yemen (Socotra Archipelago)	46.80	12.00	9.00	4.80
H. inexpectatus	IBE	IBES7735P	Oman	30.10	8.70	5.70	3.60
H. inexpectatus	ONHM	ONHM3711P	Oman	36.40	9.60	6.70	3.90
H. inexpectatus	IBE	IBES1798P	Oman	39.30	10.20	6.40	3.80
H. inexpectatus	IBE	IBES7700P	Oman	39.90	10.30	7.10	3.90
H. inexpectatus	BMNH	BMNH2008.712P	Oman	41.00	10.80	7.40	3.70
H. inexpectatus	BMNH	BMNH2008.711H	Oman	44.10	11.50	7.80	4.00
H. inintellectus	MSNPV	TR24-7	Yemen (Socotra Archipelago)	45.40	13.20	9.60	5.90
H. inintellectus	MSNPV	TR24-4	Yemen (Socotra Archipelago)	52.90	14.00	10.60	6.80
H. inintellectus	MCCI	R1441	Yemen (Socotra Archipelago)	52.00	14.30	11.20	7.30
H. inintellectus	MCCI	R1469	Yemen (Socotra Archipelago)	51.30	14.40	10.90	6.90
H. inintellectus	MCCI	R1471	Yemen (Socotra Archipelago)	56.90	14.40	11.80	7.30
H. inintellectus	MSNPV	TR24-5	Yemen (Socotra Archipelago)	51.80	15.00	10.60	6.30
H. inintellectus	MCCI	R1437	Yemen (Socotra Archipelago)	57.50	15.60	11.70	7.60
H. inintellectus	MSNPV	TR24-3	Yemen (Socotra Archipelago)	59.50	16.70	13.00	7.30
H. isolepis	NMP	NMP 74447	Somalia	34.62	?	?	?
H. jumailiae	NMP	NMP6V 74819	Yemen	40.08	10.50	8.41	5.04
H. jumailiae	BMNH	BMNH1982.1144	Yemen	43.10	11.30	9.40	4.80
H. jumailiae	BMNH	BMNH1982.1143	Yemen	48.10	11.80	9.60	4.90
H. jumailiae	BMNH	BMNH1952.1.3.52	Yemen	47.40	12.50	10.10	4.80
H. jumailiae	NHM-BS	NHM-BS N41899	Yemen	46.20	12.63	9.43	3.99
H. jumailiae	BMNH	BMNH1982.1146	Yemen	46.90	13.00	9.30	4.90
H. jumailiae	NHM-BS	NHM-BS N41891	Yemen	49.85	13.21	10.81	5.25
H. jumailiae	NHM-BS	NHM-BS N41898	Yemen	47.80	13.38	10.57	4.86
H. jumailiae	BMNH	BMNH1982.1145	Yemen	54.00	13.40	10.10	5.50
H. jumailiae	NHM-BS	NHM-BS N41897	Yemen	51.36	13.55	10.80	5.17
H. jumailiae	MSNG	MSNG-YEM02	Yemen	47.47	13.61	11.99	7.10
H. jumailiae	NHM-BS	NHM-BS N41890	Yemen	48.49	14.11	10.38	4.91
H. jumailiae	NHM-BS	NHM-BS N41894	Yemen	50.61	14.11	10.48	5.23
H. jumailiae	NHM-BS	NHM-BS N41893	Yemen	49.80	14.13	11.08	5.80
H. jumailiae	NHM-BS	NHM-BS N41788	Yemen	50.92	14.30	10.84	5.17
H. jumailiae	MCCI	R814	Yemen	54.21	14.66	13.79	6.31

H. lavadeserticus	NMP	NMP6V 35540/4	Syria	33.62	9.25	6.77	3.80
H. lavadeserticus	NMP	NMP6V 74049/5	Syria	36.53	10.60	7.58	4.39
H. lavadeserticus	NMP	NMP6V 35540/1	Syria	45.81	11.65	8.82	5.00
H. lavadeserticus	NMP	NMP6V 74049/2	Syria	42.19	11.77	9.44	5.53
H. lavadeserticus	NMP	NMP6V 74049/4	Syria	45.80	11.96	9.58	5.62
H. lavadeserticus	NMP	NMP6V 34831/1	Syria	47.33	12.06	8.89	4.48
H. lavadeserticus	NMP	NMP6V 74049/3	Syria	46.64	12.07	8.55	5.00
H. lavadeserticus	NMP	NMP6V 35540/3	Syria	47.24	12.07	8.85	5.09
H. lavadeserticus	NMP	NMP6V 35540/2	Syria	47.19	12.22	7.85	4.28
H. lavadeserticus	NMP	NMP6V 74049/1	Syria	49.57	12.38	9.45	3.79
H. lemurinus	IBE	IBES8059	Oman	46.60	13.90	11.10	6.80
H. lemurinus	IBE	IBES8058	Oman	63.60	16.80	12.80	7.90
H. lemurinus	NMP	NMP6V 74801/2	Yemen	47.66	13.75	10.70	6.91
H. lemurinus	NMP	NMP6V 74801/1	Yemen	58.98	16.11	12.65	7.63
H. luqueorum	IBE	IBES6056	Oman	59.00	15.90	11.40	5.80
H. luqueorum	BMNH	BMNH1975.916P	Oman	67.00	18.40	14.20	9.40
H. luqueorum	BMNH	BMNH2008.710	Oman	73.00	18.50	13.60	8.40
H. luqueorum	IBE	IBES8068P	Oman	76.50	19.20	14.00	9.10
H. luqueorum	BMNH	BMNH1971.41P	Oman	69.00	19.40	13.50	9.10
H. luqueorum	IBE	IBES7771P	Oman	76.40	19.80	14.80	8.20
H. luqueorum	BMNH	BMNH2005.1659P	Oman	75.00	20.00	15.00	8.50
H. luqueorum	BMNH	BMNH2005.1658P	Oman	84.70	21.20	16.30	10.30
H. luqueorum	BMNH	BMNH2005.1660H	Oman	80.40	21.30	15.90	9.90
H. luqueorum	ONHM	ONHM3705P	Oman	85.00	21.30	16.70	10.00
H. luqueorum	IBE	IBES7155	Oman	82.10	21.70	16.50	10.20
H. luqueorum	BMNH	BMNH1980.558P	Oman	88.00	23.00	18.00	10.90
H. macropholis	TMHC	TMHC2012.01.032	Ethiopia	50.84	13.05	10.79	6.12
H. macropholis	TMHC	TMHC2012.01.033	Ethiopia	51.53	13.95	10.76	6.01
H. macropholis	TMHC	TMHC2012.01.034	Ethiopia	55.62	14.50	11.75	6.11
H. macropholis	TMHC	TMHC2012.01.035	Ethiopia	54.60	15.08	11.91	6.78
H. macropholis	CAS	CAS 130234	Kenya	40.41	10.88	9.13	5.39
H. macropholis	CAS	CAS 130538	Kenya	42.18	11.25	9.33	5.70
H. macropholis	CAS	CAS130235	Kenya	41.02	11.60	9.31	5.10
H. macropholis	CAS	CAS 130513	Kenya	44.27	11.63	10.05	5.27
H. macropholis	CAS	CAS 130515	Kenya	44.21	11.91	9.32	5.26
H. macropholis	CAS	CAS130540	Kenya	46.39	12.51	10.63	6.09
H. macropholis	CAS	CAS130539	Kenya	49.82	12.73	10.46	6.71
H. macropholis	CAS	CAS146969	Kenya	43.75	12.79	10.42	5.75
H. macropholis	CAS	CAS 130517	Kenya	46.80	13.00	10.33	5.82
H. macropholis	CAS	CAS122330	Kenya	52.49	13.27	10.58	6.13
H. macropholis	CAS	CAS 130094	Kenya	47.92	13.28	10.97	6.00
H. macropholis	CAS	CAS140288	Kenya	49.12	13.39	10.90	6.81
H. macropholis	CAS	CAS130514	Kenya	49.01	13.40	11.02	6.80
H. macropholis	CAS	CAS122233	Kenya	51.74	13.46	11.15	6.35
H. macropholis	CAS	CAS130516	Kenya	52.67	14.11	11.57	7.20

H. macropholis	CAS	CAS 146970	Kenya	50.62	14.44	10.19	5.82
H. macropholis	CAS	CAS 140285	Kenya	55.14	14.47	11.79	6.37
H. macropholis	CAS	CAS130231	Kenya	55.61	14.57	12.93	7.22
H. macropholis	CAS	CAS130232	Kenya	55.08	15.39	12.98	7.62
H. macropholis	CAS	CAS 140284	Kenya	55.01	15.44	12.57	7.07
H. macropholis	CAS	CAS140287	Kenya	58.12	16.56	13.55	8.42
H. macropholis	CAS	CAS140286	Kenya	64.64	17.75	15.67	9.27
H. macropholis	MSNG	MSNG29102c	Somalia	44.52	12.40	9.82	5.71
H. macropholis	MSNM	MSNM198	Somalia	49.43	13.59	11.40	6.76
H. macropholis	MSNG	MSNG29102d	Somalia	48.36	13.97	10.80	6.50
H. macropholis	MSNG	MSNG29201	Somalia	52.43	14.10	10.27	5.76
H. macropholis	MSNM	MSNM337	Somalia	48.43	14.26	10.66	6.56
H. macropholis	MSNG	MSNG29102a	Somalia	57.87	14.28	13.25	8.29
H. macropholis	CAS	CAS153459	Somalia	53.90	14.30	11.48	6.60
H. macropholis	MSNG	MSNG29102b	Somalia	49.74	14.35	11.28	6.79
H. macropholis	CAS	CAS 158935	Somalia	54.00	14.55	11.89	6.66
H. macropholis	MZUF	MZUF10813	Somalia	54.78	14.63	11.91	7.49
H. macropholis	MZUF	MZUF24659	Somalia	53.72	14.76	12.94	8.15
H. macropholis	MZUF	MZUF24467	Somalia	57.20	15.23	12.99	7.70
H. macropholis	MSNG	MSNG28567c	Somalia	56.94	15.29	12.76	7.89
H. macropholis	MZUF	MZUF5242	Somalia	56.93	15.44	11.23	7.13
H. macropholis	MSNM	MSNM336	Somalia	56.60	15.65	12.66	7.77
H. macropholis	CAS	CAS148346	Somalia	58.95	15.65	13.78	8.46
H. macropholis	MZUF	MZUF24464	Somalia	62.23	15.65	13.15	7.67
H. macropholis	MZUF	MZUF24470	Somalia	63.31	15.75	12.87	8.63
H. macropholis	MZUF	MZUF24468	Somalia	60.68	15.76	12.96	7.26
H. macropholis	MSNM	MSNM194	Somalia	60.04	15.86	13.26	8.01
H. macropholis	MZUF	MZUF24658	Somalia	60.79	15.93	13.63	7.83
H. macropholis	MSNG	MSNG28567b	Somalia	62.89	16.11	14.40	8.36
H. macropholis	MZUF	MZUF5236	Somalia	56.95	16.24	12.77	7.71
H. macropholis	CAS	CAS 148349	Somalia	59.27	16.25	13.66	7.79
H. macropholis	MZUF	MZUF24471	Somalia	63.26	16.46	12.69	9.32
H. macropholis	MZUF	MZUF1727	Somalia	60.70	16.66	12.66	6.73
H. macropholis	CAS	CAS148347	Somalia	59.47	16.73	13.53	7.91
H. macropholis	MSNG	MSNG28567a	Somalia	60.27	16.77	13.13	7.89
H. macropholis	MZUF	MZUF1631	Somalia	58.40	16.82	13.38	8.40
H. macropholis	MZUF	MZUF21091	Somalia	63.49	16.99	13.32	8.36
H. macropholis	MZUF	MZUF1592	Somalia	61.77	17.01	14.70	7.99
H. macropholis	CAS	CAS 148348	Somalia	64.94	17.06	14.21	8.12
H. macropholis	MZUF	MZUF10824	Somalia	62.45	17.40	14.57	8.96
H. macropholis	MZUF	MZUF2988	Somalia	61.06	17.52	14.25	8.38
H. macropholis	CAS	CAS151126	Somalia	65.70	17.54	14.36	7.55
H. macropholis	MZUF	MZUF2986	Somalia	61.53	17.59	14.60	7.94
H. macropholis	CAS	CAS 148345	Somalia	66.10	17.70	14.28	8.67
H. macropholis	MSNM	MSNM335	Somalia	67.08	18.14	14.40	8.79

H. macropholis	MZUF	MZUF697	Somalia	63.53	18.20	15.11	9.29
H. macropholis	MZUF	MZUF24465	Somalia	71.77	18.24	15.94	11.47
H. macropholis	MZUF	MZUF24656	Somalia	70.72	18.30	16.70	9.85
H. macropholis	MZUF	MZUF5237	Somalia	65.52	18.31	16.03	8.95
H. macropholis	MZUF	MZUF24463	Somalia	72.63	18.46	15.60	9.64
H. macropholis	MZUF	MZUF10825	Somalia	71.59	18.52	15.41	9.20
H. macropholis	MZUF	MZUF26364	Somalia	72.91	18.80	16.54	11.37
H. macropholis	MZUF	MZUF2989	Somalia	70.30	18.84	15.27	9.19
H. macropholis	MSNM	MSNM193	Somalia	74.12	18.90	15.87	9.95
H. macropholis	MSNM	MSNM347	Somalia	71.59	19.15	16.79	9.24
H. macropholis	MZUF	MZUF24657	Somalia	66.53	19.30	17.37	10.85
H. macropholis	MSNM	MSNM344	Somalia	73.59	19.40	16.26	9.48
H. macropholis	MZUF	MZUF21092	Somalia	72.62	19.50	15.73	9.96
H. macropholis	MZUF	MZUF2987	Somalia	75.56	19.54	16.14	10.31
H. macropholis	MSNM	MSNM345	Somalia	76.58	19.86	17.59	10.30
H. macropholis	MZUF	MZUF21090	Somalia	77.99	20.66	18.17	11.99
H. macropholis	MZUF	MZUF10812	Somalia	82.20	20.70	17.57	11.83
H. macropholis	MZUF	MZUF10827	Somalia	81.13	21.28	17.66	11.90
H. macropholis	MZUF	MZUF2429	Somalia	81.30	21.95	19.36	12.54
H. mandebensis	NMP	NMP6V 74836/2	Yemen	41.49	10.55	8.54	5.08
H. masirahensis	ONHM	ONHM3710P	Oman	25.70	7.30	4.70	3.00
H. masirahensis	IBE	IBES7710P	Oman	33.50	8.30	6.10	4.10
H. masirahensis	BMNH	BMNH1975.2083P	Oman	34.30	9.20	6.30	3.60
H. masirahensis	BMNH	BMNH1975.2084P	Oman	37.60	9.50	6.90	3.90
H. masirahensis	BMNH	BMNH1975.2082P	Oman	37.20	9.90	6.30	3.50
H. masirahensis	BMNH	BMNH1975.2081P	Oman	45.00	10.70	7.60	4.70
H. masirahensis	BMNH	BMNH1975.2080H	Oman	42.10	10.80	7.90	4.70
H. mindiae	NMP	NMP6V 71323/1	Jordan	35.63	10.43	7.25	4.02
H. mindiae	NMP	NMP6V 72739/3	Jordan	37.05	10.66	7.72	4.89
H. mindiae	NMP	NMP6V 72739/2	Jordan	49.33	12.80	10.40	6.74
H. mindiae	NMP	NMP6V 72739/1	Jordan	55.67	13.88	11.53	7.14
H. mindiae	NMP	NMP6V 71323/2	Jordan	56.56	15.05	11.23	6.45
H. minutus	BMNH	BMNH1977.917	Oman	20.50	6.20	4.20	2.40
H. minutus	BMNH	BMNH1985.563	Oman	23.30	6.40	4.30	2.40
H. minutus	BMNH	BMNH1977.927	Oman	23.40	6.40	5.00	2.70
H. minutus	BMNH	BMNH1977.916	Oman	24.00	6.70	4.30	2.60
H. minutus	BMNH	BMHN1977.913	Oman	26.50	6.70	4.80	2.70
H. minutus	BMNH	BMNH1977.923	Oman	23.50	6.80	4.50	2.40
H. minutus	BMNH	BMNH1977.915	Oman	23.20	7.00	4.50	2.50
H. minutus	BMNH	BMHN1977.918	Oman	31.70	7.00	5.40	3.20
H. minutus	BMNH	BMHN1983.705	Oman	28.10	7.10	4.60	2.80
H. minutus	BMNH	BMHN1983.704	Oman	30.10	7.10	5.10	3.70
H. minutus	BMNH	BMHN1977.921	Oman	28.90	7.20	4.90	2.80
H. minutus	BMNH	BMHN1977.912	Oman	29.30	7.30	5.30	3.10
H. minutus	IBE	IBES7657	Oman	29.40	7.30	4.70	2.90

H. minutus	BMNH	BMHN1977.919	Oman	29.90	7.40	5.10	2.60
H. minutus	BMNH	BMNH1977.926	Oman	28.00	7.50	5.00	2.50
H. minutus	BMNH	BMNH1977.914	Oman	26.50	7.60	5.00	2.60
H. minutus	IBE	IBES7664	Oman	28.90	7.60	4.80	3.00
H. minutus	IBE	IBES7924	Oman	29.60	7.60	5.20	3.00
H. minutus	IBE	IBES7893	Oman	29.60	7.60	5.10	3.10
H. minutus	NMP	NMP6V 74863	Oman	29.50	7.67	5.30	3.34
H. minutus	BMNH	BMHN1977.922	Oman	30.90	7.70	5.40	3.00
H. minutus	IBE	IBES7966	Oman	31.80	7.70	5.50	3.60
H. minutus	BMNH	BMHN1977.924	Oman	32.10	7.70	5.00	2.90
H. minutus	IBE	IBES7909	Oman	29.70	7.80	5.10	2.90
H. minutus	IBE	IBES7871	Oman	28.70	7.90	4.70	2.90
H. minutus	BMNH	BMHN1977.920	Oman	31.80	8.20	5.40	3.40
H. minutus	IBE	IBES7676	Oman	32.80	8.20	6.30	3.40
H. minutus	BMNH	BMHN1977.911	Oman	32.40	8.40	5.90	3.70
H. minutus	IBE	IBES7668	Oman	30.30	8.50	6.10	3.30
H. minutus	IBE	IBES7673	Oman	33.40	8.50	5.70	3.50
H. minutus	NMP	NMP6V 74806	Yemen	31.90	7.50	5.40	3.20
H. minutus	NMP	NMP6V 74805/1	Yemen	30.50	7.60	5.40	3.10
H. minutus	NMP	NMP6V 74805/2	Yemen	31.20	7.70	5.20	3.40
H. modestus	Gunther (1894)		Kenya	45.00	?	?	?
H. montanus	NMP	NMP6V 74802	Yemen	42.38	11.97	8.29	5.28
H. montanus	NHM-BS	NHM-BS N41842	Yemen	45.15	12.69	9.17	4.54
H. montanus	NHM-BS	NHM-BS N41779	Yemen	48.53	13.01	10.09	5.00
H. montanus	NHM-BS	NHM-BS N41844	Yemen	45.71	13.07	9.62	4.27
H. montanus	NHM-BS	NHM-BS N41815	Yemen	46.50	13.07	9.41	4.82
H. montanus	NHM-BS	NHM-BS N41798	Yemen	46.60	13.17	9.49	4.86
H. montanus	NHM-BS	NHM-BS N41818	Yemen	43.77	13.18	9.08	4.77
H. montanus	NHM-BS	NHM-BS N41790	Yemen	44.00	13.33	9.20	4.35
H. montanus	NHM-BS	NHM-BS N41766	Yemen	49.84	13.49	9.91	5.01
H. montanus	NHM-BS	NHM-BS N41853	Yemen	49.15	13.68	9.64	4.97
H. montanus	NHM-BS	NHM-BS N41752	Yemen	49.09	13.74	9.60	4.11
H. montanus	NHM-BS	NHM-BS N41768	Yemen	52.46	13.82	10.10	5.48
H. montanus	NHM-BS	NHM-BS N41846	Yemen	51.02	13.92	10.68	5.43
H. montanus	NHM-BS	NHM-BS N41773	Yemen	51.66	13.96	10.57	5.47
H. montanus	NHM-BS	NHM-BS N41854	Yemen	50.21	14.05	9.61	4.76
H. montanus	NHM-BS	NHM-BS N41833	Yemen	51.27	14.07	9.72	5.18
H. montanus	NHM-BS	NHM-BS N41797	Yemen	50.28	14.10	11.26	5.34
H. montanus	NHM-BS	NHM-BS N41839	Yemen	54.09	14.37	10.98	6.06
H. montanus	NHM-BS	NHM-BS N41823	Yemen	52.65	14.52	10.13	4.69
H. montanus	NHM-BS	NHM-BS N41851	Yemen	51.73	14.55	11.84	6.04
H. montanus	NHM-BS	NHM-BS N41813	Yemen	51.70	14.65	11.13	5.53
H. montanus	NHM-BS	NHM-BS N41762	Yemen	52.00	15.03	10.04	5.52
H. montanus	NHM-BS	NHM-BS N41804	Yemen	57.40	15.03	12.36	5.33
H. montanus	NHM-BS	NHM-BS N41840	Yemen	53.74	15.06	11.18	5.44

H. montanus	NHM-BS	NHM-BS N41821	Yemen	55.86	15.10	10.60	5.54
H. montanus	NHM-BS	NHM-BS N41834	Yemen	54.78	15.16	11.97	6.14
H. montanus	NHM-BS	NHM-BS N41763	Yemen	55.68	15.24	10.93	5.70
H. montanus	NHM-BS	NHM-BS N41769	Yemen	54.25	15.26	10.90	6.24
H. montanus	NHM-BS	NHM-BS N41836	Yemen	51.82	15.27	10.81	5.35
H. montanus	NHM-BS	NHM-BS N41783	Yemen	59.04	15.43	12.38	5.50
H. montanus	NHM-BS	NHM-BS N41751	Yemen	53.63	15.46	10.66	5.52
H. montanus	NHM-BS	NHM-BS N41806	Yemen	54.25	15.52	10.53	5.12
H. montanus	NHM-BS	NHM-BS N41803	Yemen	57.18	15.53	11.50	6.28
H. montanus	NHM-BS	NHM-BS N41765	Yemen	57.28	15.58	10.87	5.36
H. montanus	NHM-BS	NHM-BS N41793	Yemen	54.68	15.59	11.64	6.05
H. montanus	NHM-BS	NHM-BS N41843	Yemen	58.50	15.60	12.42	6.03
H. montanus	NHM-BS	NHM-BS N41791	Yemen	55.84	15.84	11.37	5.65
H. montanus	NHM-BS	NHM-BS N41809	Yemen	58.74	15.91	11.81	6.00
H. montanus	NHM-BS	NHM-BS N41811	Yemen	61.71	15.98	12.32	7.04
H. montanus	NHM-BS	NHM-BS N41774	Yemen	55.76	16.00	11.14	5.20
H. montanus	NHM-BS	NHM-BS N41848	Yemen	55.53	16.02	12.13	6.02
H. montanus	NHM-BS	NHM-BS N41772	Yemen	58.72	16.06	12.27	6.52
H. montanus	NHM-BS	NHM-BS N41800	Yemen	57.44	16.13	12.42	6.18
H. montanus	NHM-BS	NHM-BS N41807	Yemen	56.99	16.14	11.60	5.87
H. montanus	NHM-BS	NHM-BS N41771	Yemen	57.00	16.37	11.87	5.97
H. montanus	NHM-BS	NHM-BS N41812	Yemen	58.48	16.55	12.12	6.50
H. montanus	NHM-BS	NHM-BS N41789	Yemen	61.56	16.70	12.44	6.10
H. montanus	NHM-BS	NHM-BS N41770	Yemen	57.07	16.74	11.66	5.93
H. montanus	NHM-BS	NHM-BS N41802	Yemen	64.47	16.78	14.13	7.64
H. montanus	NHM-BS	NHM-BS N41852	Yemen	64.14	17.29	13.42	6.64
H. montanus	NHM-BS	NHM-BS N41867	Yemen	64.07	17.81	13.12	7.54
H. montanus	NHM-BS	NHM-BS N41814	Yemen	64.50	17.82	13.55	7.20
H. ophiolepis	TMHC	TMHC 2012.07.094	Ethiopia	36.55	9.24	6.27	3.68
H. oxyrhinus	MCCI	R1587	Yemen (Socotra Archipelago)	36.40	10.00	7.30	4.60
H. oxyrhinus	MCCI	R1587	Yemen (Socotra Archipelago)	38.00	11.00	7.70	4.90
H. oxyrhinus	MCCI	R1587	Yemen (Socotra Archipelago)	38.00	11.47	8.50	5.00
H. oxyrhinus	MCCI	R1587	Yemen (Socotra Archipelago)	42.40	12.00	8.50	5.60
H. oxyrhinus	MCCI	R1587	Yemen (Socotra Archipelago)	42.60	12.00	8.50	5.60
H. oxyrhinus	MCCI	R1587	Yemen (Socotra Archipelago)	42.80	12.60	9.70	5.80
H. oxyrhinus	MCCI	R1587	Yemen (Socotra Archipelago)	44.60	12.60	9.70	6.00
H. oxyrhinus	MCCI	R1587	Yemen (Socotra Archipelago)	48.30	12.60	10.00	6.40
H. oxyrhinus	MCCI	R1587	Yemen (Socotra Archipelago)	47.86	12.90	9.80	6.40
H. pauciporosus	CAS	CAS227510	Somalia	57.08	14.86	11.55	7.09
H. pauciporosus	CAS	CAS227511	Somalia	58.71	15.34	12.33	7.31
H. pauciporosus	MZUF	MZUF6245	Somalia	60.97	16.58	13.22	7.75
H. paucituberculatus	IBE	IBES7994	Oman	25.90	7.00	5.00	2.80
H. paucituberculatus	BMNH	BMNH1977.942P	Oman	29.90	7.70	5.40	2.90
H. paucituberculatus	BMNH	BMNH1977.944P	Oman	30.10	7.80	5.30	3.00
H. paucituberculatus	ONHM	ONHM3709P	Oman	32.80	8.20	5.70	3.40

H. paucituberculatus	IBE	IBES7930	Oman	33.80	8.20	6.40	3.30
H. paucituberculatus	IBE	IBES7902	Oman	34.30	8.20	6.20	3.40
H. paucituberculatus	BMNH	BMNH1977.931P	Oman	33.60	8.40	6.10	4.10
H. paucituberculatus	BMNH	BMNH1977.935H	Oman	33.50	8.50	6.10	3.60
H. paucituberculatus	IBE	IBES8004	Oman	35.40	8.90	5.90	3.50
H. paucituberculatus	BMNH	BMNHNH1977.937P	Oman	33.50	9.00	6.40	3.40
H. paucituberculatus	BMNH	BMNH1977.936P	Oman	36.50	9.10	6.20	3.60
H. paucituberculatus	BMNH	BMNH1977.930P	Oman	38.40	9.40	7.00	3.90
H. persicus	BMNH	BMNH1971.1384	Bahrain	49.70	11.00	9.40	5.80
H. persicus	BMNH	BMNH1971.1142	Bahrain	55.20	12.90	10.50	6.70
H. persicus	BMNH	BMNH1971.1386	Bahrain	52.50	13.20	10.30	5.70
H. persicus	BMNH	BMNH1975.977	Bahrain	57.40	13.50	10.60	6.40
H. persicus	BMNH	BMNH1971.1383	Bahrain	61.20	14.00	12.50	7.60
H. persicus	BMNH	BMNH1971.31	Bahrain	60.60	15.10	11.90	6.90
H. persicus	BMNH	BMNH1971.1140	Bahrain	64.70	16.30	11.00	6.80
H. persicus	BMNH	BMNH1971.26	Bahrain	63.90	16.80	13.70	8.30
H. persicus	NMP	NMP6V 74807/1	Iran	47.29	13.04	8.76	5.14
H. persicus	NMP	NMP6V 74807/2	Iran	59.55	13.39	11.15	6.11
H. persicus	NMP	NMP6V 74807/3	Iran	63.19	16.23	13.03	7.75
H. persicus	NMP	NMP6V 74807/4	Iran	71.11	19.98	13.16	7.60
H. persicus	BMNH	BMNH1961.1504	Iraq	36.90	9.20	7.20	4.90
H. persicus	BMNH	BMNH1921.3.29.1	Iraq	63.50	16.00	12.50	7.60
H. persicus	BMNH	BMNH85.7.11.3	Pakistan	57.10	12.00	11.00	6.60
H. persicus	BMNH	BMNH85.7.11.2	Pakistan	58.00	15.00	11.50	7.50
H. persicus	BMNH	BMNH76.10.28.8	Pakistan	66.10	16.10	14.40	9.60
H. persicus	BMNH	BMNH1983.1439	Saudi Arabia	50.10	12.70	9.43	5.65
H. persicus	BMNH	BMNH1983.1440	Saudi Arabia	47.40	13.40	9.10	5.80
H. pumilio	MCCI	R1513	Yemen (Socotra Archipelago)	20.00	5.60	4.20	2.80
H. pumilio	MCCI	R1512-2	Yemen (Socotra Archipelago)	22.80	6.50	4.40	2.80
H. pumilio	MCCI	R1484-1	Yemen (Socotra Archipelago)	26.40	6.70	4.80	3.50
H. pumilio	MCCI	R1512-1	Yemen (Socotra Archipelago)	25.60	6.90	4.60	3.50
H. pumilio	MCCI	R1443	Yemen (Socotra Archipelago)	29.50	7.40	4.90	3.20
H. pumilio	MCCI	R1514	Yemen (Socotra Archipelago)	30.10	7.40	5.00	3.40
H. pumilio	MCCI	R1484-2	Yemen (Socotra Archipelago)	25.50	7.60	5.20	3.40
H. robustus	BMNH	BMNH1959.1.5.11	Eritrea	46.50	11.00	9.20	5.70
H. robustus	TMHC	TMHC2012.07.093	Ethiopia	46.12	11.01	8.66	5.09
H. robustus	TMHC	TMHC2012.07.092	Ethiopia	43.70	11.34	8.63	5.22
H. robustus	BMNH	BMNH84.7.25.7	India	43.60	10.50	8.00	4.90
H. robustus	BMNH	BMNH84.7.25.6	India	44.90	11.30	8.30	5.10
H. robustus	NMP	NMP6V 74820	Iran	32.70	8.67	6.33	4.19
H. robustus	TMHC	TMHC 2012.06.068	Kenya	37.04	9.60	6.97	3.72
H. robustus	CAS	CAS130512	Kenya	43.44	11.69	8.85	5.43
H. robustus	BMNH	BMNH1977.37	Oman	31.20	7.90	6.20	3.90
H. robustus	NMP	NMP6V 74867/3	Oman	37.09	9.21	7.27	4.23
H. robustus	NMP	NMP6V 74867/1	Oman	37.38	9.63	7.37	4.24

H. robustus	BMNH	BMNH1975.1032	Oman	42.00	10.10	7.70	4.80
H. robustus	NMP	NMP6V 74867/2	Oman	41.85	10.38	8.32	4.47
H. robustus	NMP	NMP6V 74869/7	Oman	41.90	10.42	8.18	4.52
H. robustus	BMNH	BMNH1977.36	Oman	42.40	10.50	7.50	5.20
H. robustus	NMP	NMP6V 74869/1	Oman	42.91	10.57	8.15	4.53
H. robustus	NMP	NMP6V 74870/2	Oman	47.97	10.73	8.76	4.29
H. robustus	NMP	NMP6V 74869/5	Oman	45.45	10.99	7.96	4.82
H. robustus	NMP	NMP6V 74869/2	Oman	43.52	11.01	7.97	4.47
H. robustus	NMP	NMP6V 74869/6	Oman	47.37	11.29	8.52	4.88
H. robustus	NMP	NMP6V 74869/4	Oman	47.35	11.47	8.74	4.91
H. robustus	NMP	NMP6V 74870/1	Oman	47.27	11.58	8.93	5.05
H. robustus	BMNH	BMNH1985.564	Oman	44.30	12.00	8.10	5.50
H. robustus	BMNH	BMNH1973.439	Pakistan	36.20	8.70	6.90	4.20
H. robustus	BMNH	BMNH1973.438	Pakistan	39.50	9.20	7.90	4.40
H. robustus	BMNH	BMNH83.3.26.6	Pakistan	44.00	9.40	7.90	4.80
H. robustus	BMNH	BMNH1973.440	Pakistan	38.90	9.50	7.20	4.30
H. robustus	BMNH	BMNH83.3.26.5	Pakistan	44.80	10.60	8.10	5.10
H. robustus	BMNH	BMNH1934.11.8.12	Saudi Arabia	38.00	10.00	6.70	4.50
H. robustus	BMNH	BMNH1934.11.8.11	Saudi Arabia	42.60	10.00	7.50	5.10
H. robustus	BMNH	BMNHNH1934.11.8.10	Saudi Arabia	46.20	10.40	8.90	5.00
H. robustus	BMNH	BMNH1992.200	Saudi Arabia	45.50	11.20	7.60	5.20
H. robustus	BMNH	BMNH1978.2031	Saudi Arabia	46.10	11.20	8.60	4.90
H. robustus	BMNH	BMNH1992.201	Saudi Arabia	44.40	11.40	9.00	5.30
H. robustus	BMNH	BMNH1978.910	Saudi Arabia	50.80	11.50	9.20	6.10
H. robustus	BMNH	BMNH1937.12.5.271	Somalia	41.70	10.70	8.60	5.00
H. robustus	BMNH	BMNH1937.12.5.267	Somalia	42.40	10.80	7.40	4.50
H. robustus	BMNH	BMNH1937.12.5.272	Somalia	42.80	10.90	8.60	5.10
H. robustus	BMNH	BMNH1937.12.5.270	Somalia	45.70	11.80	9.30	5.50
H. robustus	BMNH	BMNH1971.1145	United Arab Emirates	33.40	8.00	6.40	4.10
H. robustus	BMNH	BMNH1973.1844	United Arab Emirates	39.20	10.00	7.40	4.70
H. robustus	BMNH	BMNHNH1973.1842	United Arab Emirates	40.90	10.30	8.00	5.60
H. robustus	BMNH	BMNH1973.1841	United Arab Emirates	48.50	10.50	8.20	5.60
H. robustus	BMNH	BMNH1971.1144	United Arab Emirates	45.40	10.60	8.10	5.20
H. robustus	BMNH	BMNH1982.258	United Arab Emirates	45.70	10.80	8.90	5.70
H. robustus	BMNH	BMNH1971.1143	United Arab Emirates	46.40	11.70	9.00	5.40
H. robustus	BMNH	BMNH1963.673	Yemen	39.00	9.60	6.70	4.10
H. robustus	BMNH	BMNH1965.1448	Yemen	36.00	9.70	7.00	4.30
H. robustus	NMP	NMP6V 74821/1	Yemen	41.31	9.80	7.23	3.77
H. robustus	BMNH	BMNH97.11.33	Yemen	42.60	9.80	6.90	4.60
H. robustus	BMNH	BMNH1953.1.7.89	Yemen	47.40	10.00	8.60	5.56
H. robustus	BMNH	BMNH1953.1.7.88	Yemen	47.10	10.30	8.40	5.70
H. robustus	BMNH	BMNH1963.672	Yemen	45.70	10.60	8.90	5.70
H. robustus	NMP	NMP6V 74821/2	Yemen	43.35	10.65	8.55	5.47
H. robustus	BMNH	BMNH97.11.32	Yemen	44.70	10.90	8.00	5.10
H. robustus	BMNH	BMNH1956.1.6.68	Yemen	49.10	12.20	8.50	5.30

H. robustus	BMNH	BMNH97.11.31	Yemen	51.30	12.20	9.50	6.20
H. robustus	NMP	NMP6V 74829	Yemen	50.10	12.76	9.36	5.20
H. robustus	BMNH	BMNH1956.1.6.67	Yemen	54.60	13.40	9.90	6.10
H. saba	NHM-BS	NHM-BS N41914	Yemen	47.91	12.84	8.38	4.93
H. saba	NHM-BS	NHM-BS N41913	Yemen	59.05	14.46	10.00	6.19
H. saba	NHM-BS	NHM-BS N41912	Yemen	58.30	15.40	11.38	6.41
H. shihraensis	NMP	NMP6V 74817/6	Yemen	36.73	10.16	7.44	4.85
H. shihraensis	NMP	NMP6V 74817/1	Yemen	41.89	11.22	8.03	5.34
H. shihraensis	NMP	NMP6V 74817/8	Yemen	46.65	11.52	8.74	5.32
H. shihraensis	NMP	NMP6V 74816	Yemen	42.10	12.01	8.87	4.96
H. shihraensis	NMP	NMP6V 74817/3	Yemen	47.36	12.78	8.90	5.89
H. shihraensis	NMP	NMP6V 74817/4	Yemen	47.88	12.90	8.89	5.89
H. shihraensis	NMP	NMP6V 74817/9	Yemen	47.45	13.01	9.26	6.32
H. shihraensis	NMP	NMP6V 74817/2	Yemen	49.76	13.30	9.83	6.64
H. shihraensis	NMP	NMP6V 74817/7	Yemen	50.15	13.43	9.75	6.27
H. shihraensis	NMP	NMP6V 74817/5	Yemen	53.12	14.25	10.24	6.20
H. sinaitus	MZUF	MZUF10914	Eritrea	37.33	9.59	7.39	4.70
H. sinaitus	MSNM	MSNM524	Eritrea	42.80	10.90	7.82	5.10
H. sinaitus	MSNM	MSNM523	Eritrea	47.18	12.95	10.00	6.01
H. sinaitus	NMP	NMP6V 74810	Sudan	36.43	9.62	7.42	4.88
H. sinaitus	NMP	NMP6V 74809/3	Sudan	45.49	11.34	8.69	5.75
H. sinaitus	NMP	NMP6V 74809/4	Sudan	46.88	11.75	9.17	5.57
H. sinaitus	NMP	NMP6V 74809/1	Sudan	49.05	12.40	10.62	7.16
H. sinaitus	CAS	CAS 174021	Sudan	52.02	13.22	9.61	5.99
H. sinaitus	CAS	CAS174022	Sudan	53.81	13.40	10.38	6.48
H. sinaitus	NMP	NMP6V 74809/2	Sudan	49.14	13.54	10.31	7.15
H. sp10	NMP	JS187	Kenya	33.81	?	?	?
H. sp9	MZUF	MZUF204	Eritrea	40.13	10.76	7.81	4.75
H. sp9	MZUF	MZUF159	Eritrea	46.27	11.91	9.32	5.90
H. sp9	MZUF	MZUF12257	Ethiopia	40.89	10.87	8.11	4.41
H. sp9	TMHC	TMHC 2012.06.069	Ethiopia	41.52	11.04	8.56	5.46
H. sp9	TMHC	TMHC 2012.06.070	Ethiopia	41.58	11.23	8.35	5.11
H. sp9	TMHC	TMHC 2012.06.071	Ethiopia	42.08	11.46	8.42	5.35
H. sp9	MZUF	MZUF12260	Ethiopia	49.34	11.75	9.11	5.75
H. sp9	MZUF	MZUF12259	Ethiopia	49.00	12.24	9.40	5.00
H. sp9	TMHC	TMHC 2012.06.072	Ethiopia	48.26	12.85	10.17	6.46
H. squamulatus	NMP	NMP6V 74972	Kenya	27.47	7.24	5.39	3.83
H. squamulatus	NMP	NMP6V 74872/1	Kenya	34.81	8.61	6.48	4.27
H. squamulatus	NMP	NMP6V 74971	Kenya	35.41	8.80	6.49	3.79
H. squamulatus	NMP	NMP6V 74872/6	Kenya	37.47	9.46	7.07	3.96
H. squamulatus	TMHC	TMHC 2013.10.447	Kenya	38.25	9.55	6.83	4.76
H. squamulatus	NMP	NMP6V 74872/5	Kenya	40.32	9.88	6.88	4.89
H. squamulatus	NMP	NMP6V 74872/4	Kenya	42.00	10.35	8.02	4.49
H. squamulatus	NMP	NMP6V 74872/3	Kenya	43.17	10.81	8.21	5.40
H. squamulatus	NMP	NMP6V 74872/2	Kenya	44.57	10.84	7.79	4.98

H. turcicus	NMP	NMP6V 71587/2	Cyprus	40.17	9.87	8.76	5.58
H. turcicus	NMP	NMP6V 71587/3	Cyprus	41.25	10.68	8.67	4.30
H. turcicus	NMP	NMP6V 71587/1	Cyprus	48.23	12.22	9.20	5.46
H. turcicus	NMP	NMP6V 71592/1	Cyprus	49.63	12.65	9.91	6.33
H. turcicus	NMP	NMP6V 71592/2	Cyprus	52.74	12.75	10.24	6.16
H. turcicus	NMP	NMP6V 71056	Egypt	50.17	12.29	9.59	6.38
H. turcicus	NMP	NMP6V 74167	Greece	55.69	13.08	10.49	6.42
H. turcicus	NMP	NMP6V 70667	Greece	50.89	13.23	9.63	5.71
H. turcicus	NMP	NMP6V 74050	Greece	54.06	13.36	10.30	6.55
H. turcicus	NMP	NMP6V 72073	Greece	56.20	13.65	11.46	6.93
H. turcicus	NMP	NMP6V 70269	Italy	50.64	12.48	10.54	6.19
H. turcicus	NMP	NMP6V 72497	Syria	37.25	10.36	7.56	4.92
H. turcicus	NMP	NMP6V 34747	Syria	42.22	10.57	7.76	4.58
H. turcicus	NMP	NMP6V 34749	Syria	42.77	11.62	8.87	5.25
H. turcicus	NMP	NMP6V 74046/2	Syria	47.79	12.03	9.42	5.10
H. turcicus	NMP	NMP6V 74046/1	Syria	53.19	12.27	9.12	6.07
H. turcicus	NMP	NMP6V 34748/2	Syria	47.22	12.35	10.09	5.60
H. turcicus	NMP	NMP6V 74131/1	Syria	51.03	12.55	10.98	6.06
H. turcicus	NMP	NMP6V 34748/1	Syria	48.50	12.78	10.06	5.88
H. turcicus	NMP	NMP6V 74131/3	Syria	52.64	12.95	10.27	5.71
H. turcicus	NMP	NMP6V 74131/2	Syria	53.38	13.13	11.05	6.49
H. turcicus	NMP	NMP6V 34748/3	Syria	55.90	13.30	10.36	6.50
H. turcicus	NMP	NMP6V 70648/4	Turkey	26.43	7.70	5.26	3.41
H. turcicus	NMP	NMP6V 70648/3	Turkey	30.60	8.46	5.65	3.47
H. turcicus	NMP	NMP6V 74047/2	Turkey	38.18	9.51	7.16	4.03
H. turcicus	NMP	NMP6V 74047/1	Turkey	39.38	10.34	7.43	4.28
H. turcicus	NMP	NMP6V 70648/1	Turkey	45.64	11.52	9.05	5.71
H. turcicus	NMP	NMP6V 70648/2	Turkey	48.56	11.99	9.26	5.15
H. turcicus	NMP	NMP6V 73626/3	Turkey	42.58	12.29	8.37	5.23
H. turcicus	NMP	NMP6V 73626/2	Turkey	46.49	12.85	9.20	5.53
H. turcicus	NMP	NMP6V 73626/1	Turkey	52.55	13.61	10.19	6.50
H. ulii	NMP	NMP6V 74833/1	Yemen	36.77	9.67	8.01	4.50
H. ulii	NMP	NMP6V 74833/2	Yemen	40.36	10.00	8.63	5.24
H. ulii	NMP	NMP6V 74834/1	Yemen	39.39	10.02	8.13	4.40
H. ulii	NMP	NMP6V 74831/1	Yemen	40.73	10.12	8.15	4.88
H. yerburii	MSNG	MSNG-YEM01	Yemen	43.57	12.43	10.00	5.50
H. yerburii	MSNG	MSNG-YEM06	Yemen	45.75	12.70	9.91	5.52
H. yerburii	NHM-BS	NHM-BS N41869	Yemen	43.59	12.75	9.40	5.10
H. yerburii	NHM-BS	NHM-BS N41858	Yemen	54.04	13.36	11.28	5.94
H. yerburii	NMP	NMP6V 74828/3	Yemen	51.40	13.50	11.24	5.85
H. yerburii	NHM-BS	NHM-BS N41871	Yemen	46.41	13.55	9.13	5.06
H. yerburii	NHM-BS	NHM-BS N41860	Yemen	43.63	13.58	10.10	5.42
H. yerburii	NMP	NMP6V 74825/2	Yemen	50.65	13.59	11.15	6.31
H. yerburii	MSNG	MSNG-YEM05	Yemen	52.54	14.13	11.92	6.21
H. yerburii	NMP	NMP6V 74825/1	Yemen	54.19	14.41	11.18	5.75

H. yerburii	BMNH	BMNH1945.12.18.12	Yemen	54.00	14.50	10.80	7.00
H. yerburii	NMP	NMP6V 74826	Yemen	57.76	14.59	11.78	6.55
H. yerburii	NMP	NMP6V 74828/2	Yemen	54.88	14.67	12.23	6.80
H. yerburii	NMP	NMP6V 74827/4	Yemen	58.12	14.67	12.06	7.20
H. yerburii	NMP	NMP6V 74822/5	Yemen	54.98	14.89	11.08	6.17
H. yerburii	NMP	NMP6V 74823/1	Yemen	59.86	15.00	12.83	6.67
H. yerburii	NHM-BS	NHM-BS N41879	Yemen	50.12	15.01	11.08	6.26
H. yerburii	NHM-BS	NHM-BS N41857	Yemen	57.33	15.17	11.21	6.06
H. yerburii	NHM-BS	NHM-BS N41875	Yemen	55.68	15.24	11.17	6.09
H. yerburii	NHM-BS	NHM-BS N41885	Yemen	53.70	15.31	11.03	6.34
H. yerburii	BMNH	BMNH95.5.23.9	Yemen	67.60	15.40	14.60	9.10
H. yerburii	NHM-BS	NHM-BS N41883	Yemen	55.09	15.46	11.17	5.75
H. yerburii	NHM-BS	NHM-BS N41866	Yemen	54.97	15.52	11.67	6.38
H. yerburii	NMP	NMP6V 74828/1	Yemen	60.18	15.53	12.76	7.66
H. yerburii	NHM-BS	NHM-BS N41887	Yemen	55.82	15.55	11.07	5.56
H. yerburii	NHM-BS	NHM-BS N41880	Yemen	59.68	15.57	11.24	6.50
H. yerburii	NMP	NMP6V 74823/3	Yemen	59.20	15.60	12.11	6.48
H. yerburii	NMP	NMP6V 74827/3	Yemen	59.04	15.68	12.83	6.32
H. yerburii	NHM-BS	NHM-BS N41873	Yemen	57.19	15.87	11.83	6.58
H. yerburii	NHM-BS	NHM-BS N41877	Yemen	58.88	15.87	12.04	6.97
H. yerburii	NHM-BS	NHM-BS N41884	Yemen	57.94	15.94	11.10	6.07
H. yerburii	NMP	NMP6V 74823/2	Yemen	61.59	16.23	13.25	6.18
H. yerburii	NHM-BS	NHM-BS N41876	Yemen	56.93	16.24	12.17	6.50
H. yerburii	BMNH	BMNH1987.846	Yemen	62.70	16.30	12.10	8.00
H. yerburii	NHM-BS	NHM-BS N41863	Yemen	59.94	16.39	11.53	6.58
H. yerburii	BMNH	BMNH95.5.23.8	Yemen	61.50	16.40	12.40	8.30
H. yerburii	NHM-BS	NHM-BS N41872	Yemen	56.48	16.50	11.38	6.87
H. yerburii	NHM-BS	NHM-BS N41862	Yemen	59.26	16.58	13.12	7.29
H. yerburii	NHM-BS	NHM-BS N41882	Yemen	58.93	16.63	12.72	6.20
H. yerburii	NHM-BS	NHM-BS N41856	Yemen	59.09	16.70	12.13	6.20
H. yerburii	NHM-BS	NHM-BS N41864	Yemen	61.17	16.83	13.59	7.19
H. yerburii	NMP	NMP6V 74827/2	Yemen	65.05	16.90	14.91	8.18
H. yerburii	BMNH	BMNH95.11.27.3	Yemen	63.60	17.00	13.10	7.90
H. yerburii	NHM-BS	NHM-BS N41861	Yemen	62.10	17.06	13.10	7.29
H. yerburii	NHM-BS	NHM-BS N41859	Yemen	59.93	17.18	12.58	7.28
H. yerburii	NHM-BS	NHM-BS N41886	Yemen	60.90	17.45	13.06	7.27
H. yerburii	NMP	NMP6V 74827/1	Yemen	63.82	17.47	13.39	8.15
H. yerburii	NHM-BS	NHM-BS N41881	Yemen	65.16	17.53	13.83	7.49
H. yerburii	NHM-BS	NHM-BS N41888	Yemen	67.17	17.85	14.48	8.01
H. yerburii	NMP	NMP6V 74824/1	Yemen	67.04	18.06	14.71	8.52
H. yerburii	NHM-BS	NHM-BS N41868	Yemen	74.94	19.93	15.55	8.06

References in Table S2:

Gunther, A. 1894 Report on the collection of reptiles and fishes made by Dr. JW Gregory during his expedition to Mount Kenia. In *Proc. Zool. Soc. Lond*, pp. 91.

Lanza, B. 1978 on some new or interesting east african amphibians and reptiles: pubblicazioni del centro di studio per la faunistica ed ecologia tropicali del CNR: CL. *Monit. Zool. Ital. Suppl.* **10**, 229–297.

Table S3. Results of the phylogenetic ANOVA (on body size) and MANOVA (on head proportions) on the summary tree and the 1,500 trees, using different groupings for island and continental categories. The F-values for ANOVA and the Wilks lambda for MANOVA are given in each case with their associated *p*-values.

grouping	Trait	Statistic - Summary tree	p-value - Summary	Statistic – 1500 trees	p-value - 1500 trees
mainland vs islands	SVL (F - value)	1.20	0.53	0.69	0.54-0.65
mainland vs islands	Head (Wilks lambda)	0.79	0.35	0.79-0.80	0.22-0.45
mainland vs Socotra vs Abd al Kuri	SVL (F - value)	0.69	0.60	1.20	0.48-0.62
mainland vs Socotra vs Abd al Kuri	Head (Wilks lambda)	0.80	0.70	0.79-0.80	0.58-0.80
